# Device-Based Sympathetic Nerve Regulation for Cardiovascular Diseases

**DOI:** 10.3389/fcvm.2021.803984

**Published:** 2021-12-09

**Authors:** Le Li, Zhao Hu, Yulong Xiong, Yan Yao

**Affiliations:** National Center for Cardiovascular Diseases, Peking Union Medical College, Chinese Academy of Medical Sciences, Fu Wai Hospital, Beijing, China

**Keywords:** sympathetic nervous system, device-based therapy, denervation, hypertension, heart failure, arrythmia

## Abstract

Sympathetic overactivation plays an important role in promoting a variety of pathophysiological processes in cardiovascular diseases (CVDs), including ventricular remodeling, vascular endothelial injury and atherosclerotic plaque progression. Device-based sympathetic nerve (SN) regulation offers a new therapeutic option for some CVDs. Renal denervation (RDN) is the most well-documented method of device-based SN regulation in clinical studies, and several large-scale randomized controlled trials have confirmed its value in patients with resistant hypertension, and some studies have also found RDN to be effective in the control of heart failure and arrhythmias. Pulmonary artery denervation (PADN) has been clinically shown to be effective in controlling pulmonary hypertension. Hepatic artery denervation (HADN) and splenic artery denervation (SADN) are relatively novel approaches that hold promise for a role in cardiovascular metabolic and inflammatory-immune related diseases, and their first-in-man studies are ongoing. In addition, baroreflex activation, spinal cord stimulation and other device-based therapies also show favorable outcomes. This review summarizes the pathophysiological rationale and the latest clinical evidence for device-based therapies for some CVDs.

## Introduction

Sympathetic hyperactivation plays a key role in promoting numerous pathophysiological processes in CVDs, including ventricular remodeling, vascular endothelial injury and atherosclerotic plaque progression (1). Inhibiting overexcited sympathetic nerve (SN) with drugs such as beta-blocker, angiotensin-converting enzyme inhibitor (ACEI) or angiotensin receptor blocker (ARB) may improve the prognosis of CVDs (2). The therapeutic effects on some diseases, however, are still unsatisfactory due to issues of drug efficacy, safety and compliance. The treatment of CVDs by regulating sympathetic activity has become a hot research topic in recent years, providing new therapeutic options for a variety of CVDs by maintaining the balance of the autonomic nervous system (3). Based on the effect of the intervention, device-based SN regulation can be divided into two categories: sympathetic inhibition and sympathetic stimulation. The former one, including renal denervation (4), pulmonary denervation (5), etc., can destroy the target nerve fibers by means of catheter interventions using energy such as radiofrequency inside the vessels rich in sympathetic nerve fiber distribution. The latter one incorporates carotid sinus electrical stimulation (6) and spinal cord stimulation (7), in which stimulation signals are artificially delivered to the target nerve by means of a pulse transmitter to modulate sympathetic nerve activity.

**Figure 1:**
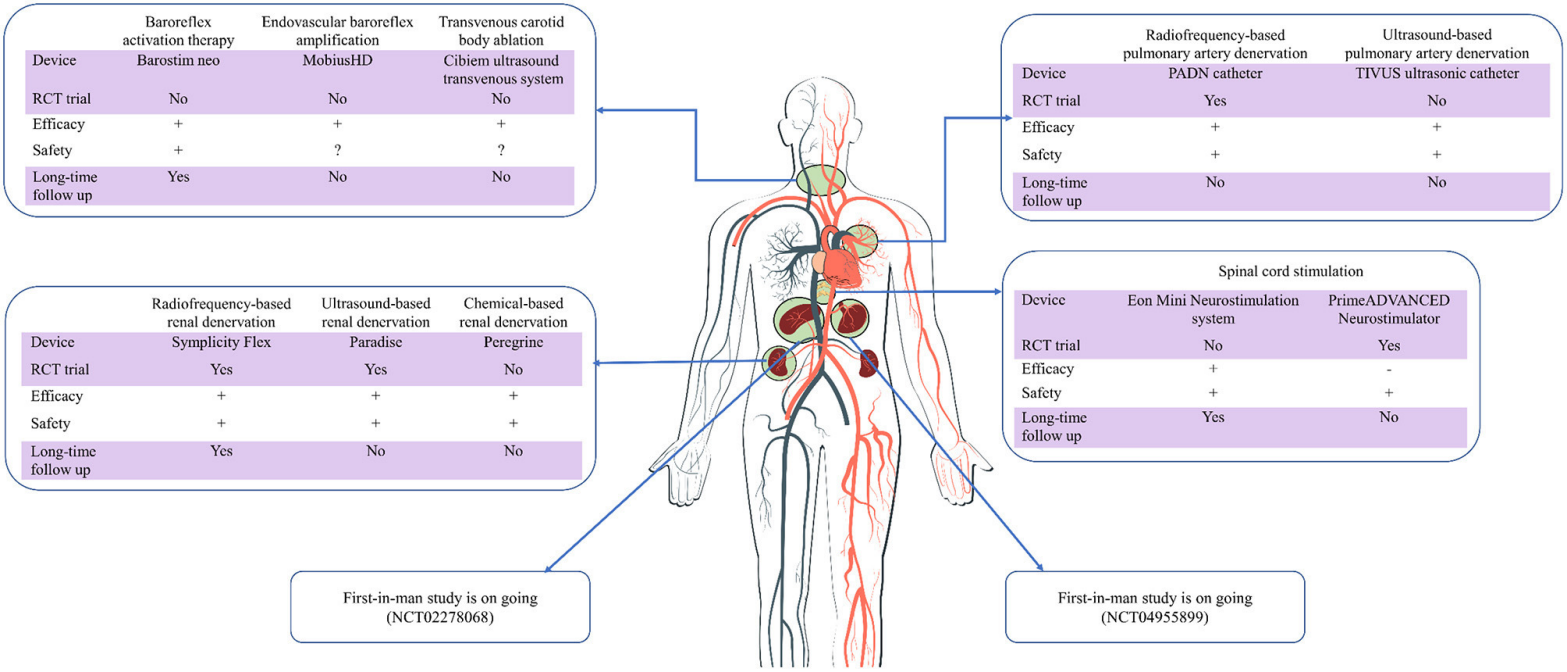
Current evidence of device-based sympathetic nerve regulation in cardiovascular diseases. RCT, randomized controlled trial; Long-time, longer than 1 year; +/–/?, positive/negative/uncertain results.

Although the precise mechanisms of action of sympathetic device modulation are not fully understood, there are research studies to support its value in common CVDs such as hypertension (8), arrhythmias (9), and heart failure (HF) (10) (Figure 1). This article will provide a review of the possible pathophysiological rationale for the application of device-based SN modulation in CVD, the latest clinical research evidence, current controversies, and future perspectives.

## Renal Denervation

### Anatomy and Mechanism

Anatomically, the kidney is innervated by both sensory afferent fibers, sympathetic efferent fibers, and parasympathetic efferent fibers, creating a bidirectional connection with the central nervous system (CNS) (11). In traditional opinion, renal SN emanate from spinal nerves, mostly *via* ventral ganglion perforators, and to a lesser extent *via* ganglia from perforators in the adrenal gland and abdominal aorta, innervating the kidney in a basket-weave structure and encircling the outer membrane of the renal artery (12). Afferent fibers of the renal nerves sense mechanical and chemical changes, and transmit these feedback signals to CNS, which consequently issues efferent signals to regulate renal vascular tone and volume homeostasis (13). Functionally, renal afferent fibers are mainly divided into pressor and depressor fibers, and the combined effect of the two different afferent fibers can lead to either an elevation or a reduction of the sympathetic tone, causing an increase or decrease in blood pressure (BP) (14). According to the change in BP after endovascular electrical stimulation, Fudim et al. (15) classified the nerve sites around the renal artery as hot spot (increased BP), cold spot (decreased BP), and neutral spot (unchanged BP). The same bundle may contain different types or functions of nerve fibers, whether RDN could reduce BP depends upon which nerve fibers are dominant at this particular site. The renal sympathetic efferent fibers innervate the renal vascular system and various parts of the renal unit, regulating renal blood flow, sodium-water reabsorption, and the release of renin and prostaglandins, affecting the function of the renal and cardiovascular systems. The anatomy and function of renal parasympathetic efferent fibers have been debated for many years. Although a study using neural tracer technology has not found a direct connection between the kidney and pre-ganglionic fibers of the vagus nerve (16), human autopsy findings have confirmed the presence of parasympathetic fiber distribution around the renal artery (17). Nevertheless, the physiological function of renal parasympathetic nerves remains to be explored.

As mentioned previously, endovascular renal electrical stimulation (RNS) could identify positive nerve-enriched area, which helps to guide selective RDN and enhance the efficacy of RDN (18). An animal study by Liu et al. (19) showed that RNS-guided RDN was more effective in lowering BP than traditional RDN (28.6 ± 6.7 vs. 14.7 ± 6.1 mm Hg, *p* = 0.002), and tyrosine hydroxylase staining confirmed that RNS-guided RDN achieved more complete denervation. In addition, nowadays, RDN lacks a valid ablation endpoint, and operators often fail to know whether the procedure is successful, which limits the widespread use of RDN. RNS may help to distinguish the complete denervation, and changes in BP after RNS may serve as an effective ablation endpoint (20). Hoogerwaard et al. (21) reported that, after 10-month follow-up, patients without residual RNS-induced BP response had a significant lower 24-h systolic BP compared to the patients with residual RNS-induced BP response (126 ± 4 vs. 135 ± 10 mmHg, *p* = 0.04). This finding may conduce to solve the issue of “blind RDN,” and patients who still respond to RNS after RDN may be advised to receive the second RDN.

The anatomical evidence regarding the innervation of the renal artery has been recently updated. Sakakura et al. (22) performed autopsies on 20 deceased patients, and a total of 10,329 nerves were identified in 300 pathological sections of the whole segment of the renal artery. They found that the SN distribution of the renal artery was characterized by more proximal and middle but fewer distal segments, more ventral but fewer dorsal segments, and more efferent fibers but fewer afferent fibers. The measurement of the distance from the nerve fibers to the lumen using morphological analysis software also revealed that the distance gradually decreased from proximal to distal segments of the artery, which is an important guide to the specific ablation sites selection and ablation parameters of the RDN and helps to further improve the results of the procedure. However, Sakakura et al. did not include branch arteries and renal artery variants (polar arteries, etc.) in their study, and Garcia-Touchard et al. (23) explored the anatomy in a larger size of autopsy samples, and performed autopsy of 60 renal arteries from 30 deceased patients. They found that the traditional basket weave structure of the renal SN was uncommon in the population (17%), whereas in most cases the renal SN bypassed the main renal artery and was directly connected to the branch or polar arteries (73% in the right kidney and 53% in the left kidney), which may partly account for the undesired BP reduction after RDN of the main renal artery in some patients (24). Therefore, ablation of the renal artery branches and the polar arteries could theoretically further improve the BP lowering effect, and the clinical study by Fengler et al. seems to support this view (25).

However, the specific mechanisms of action of RDN to benefit CVD are currently unclear. Locally, the efferent fibers of the renal SN are mostly adrenergic and mediate renal vasoconstriction, decreased urinary sodium excretion, and increased renin release through the release of norepinephrine (13). RDN blocks the activation of renal sympathetic efferent fibers, resulting in increased sodium-water excretion, decreased renin release, reduced renin-angiotensin-aldosterone (RAAS) system activity and downstream cascade effects, which may be one of the antihypertensive mechanisms (26). In animal models, RDN effectively reduced renin activity and blocks norepinephrine levels (27, 28), and has been further confirmed in subsequent clinical studies (29). However, reports on the regulation of sodium-water excretion by RDN have not reached uniform conclusions (30, 31). The above studies suggest that the antihypertensive mechanisms of RDN may be multifaceted. In addition to hypotension, sympathetic modulation may be beneficial in the control of arrhythmias (32), and RDN is a promising approach (33). The animal experiment by Huang et al. showed that stimulation of perirenal SN enhanced stellate ganglion function and caused cardiac sympathetic hyperexcitability (34). Moreover, Tsai et al. (35) observed that bilateral RDN caused a reduction in brainstem uptake of ^18^FDG as well as bilateral stellate ganglion cell necrosis, causing central and peripheral sympathetic remodeling, which may be a possible reason for RDN to reduce arrhythmic events. RDN has led to a new direction for HF treatment by reducing renal and systemic sympathetic tone (36). In addition, the results of preliminary animal studies showed that RDN could reduce circulating levels of norepinephrine and angiotensin I and II (37) which are key humoral factors that promote ventricular remodeling and HF progression (38). RDN was found to inhibit enkephalinase activity (39), thereby increasing the bioavailability of natriuretic peptides and improving cardiomyocyte and vascular physiology and function (40). The above studies suggest that RDN could function by downregulating sympathetic activity and its downstream humoral factors expression.

## Clinical Evidence

### Hypertension

Over the past few decades, the hypotensive effect of surgical splanchnicectomy has been demonstrated in a large number of studies. The procedure, however, was finally abandoned after the advent of several effective antihypertensive drugs due to its association with perioperative mortality and long-term complications (41). Just over 40 years later in 2009, catheter-based RDN was introduced to control hypertension in a minimally invasive approach (42), a boom in device interventions for SN treatment of hypertension has been initiated.

SYMPLICITY HTN-1 (43) and SYMPLICITY HTN-2 (44), feasibility studies on monopolar ablation catheter-based RDN, initially validated the efficacy and safety of RDN, with significant reductions in BP in patients with resistant hypertension in both studies, and no serious complications related to manipulation or devices were observed in either study. A new light has been shed for patients with refractory hypertension and those with poor medication adherence. However, the SYMPLICITY HTN-3 study (24) published in 2014 showed that the difference of BP reduction between RDN and sham group did not reach the significance, which halted the development of RDN for a while. The study included 535 patients with resistant hypertension who were randomly assigned to the RDN group and sham-controlled group, the primary outcome was the change in BP at 6 months, the results were demonstrated negative with BP reduction 14.13 ± 23.93 mmHg and 11.74 ± 25.94 mmHg [mean difference: −2.39 mmHg (95%CI: −6.89 to 2.12, *p* = 0.26)] in RDN and control group, respectively (24). However, there were some limitations in the study design and execution, such as inclusion of patients with isolated systolic hypertension, failure to exclude secondary hypertension, medication compliance issues during follow-up, lack of standardized surgical procedures, and limited operator experience, all of which may have affected the veracity (45). To standardize the study and remove confounding factors from the results, the DENERHTN study screened 106 patients with strictly defined resistant hypertension for receiving 4 consecutive weeks of standardized triple antihypertensive therapy before randomization to reduce variability in baseline BP levels, and used a questionnaire combined with pharmacological testing of biological samples to accurately assess subjects' medication, after 6 months follow-up, the results showed that the RDN was more effective in lowering BP than pharmacotherapy alone, with a difference of −5.9 mmHg (95% CI: −11.3 to −0.5, *p* = 0.033) between groups, reaffirming the effectiveness of RDN through a good trial design (46).

The above studies all used monopolar ablation catheters, which may suffer from insufficient breadth and depth of ablation (45). The new generation of SPYRAL multipolar ablation catheter (Medtronic, Galway, Ireland) helps to solve this problem, and a series more rigorous randomized, sham-operated controlled clinical trials according to the design method and surgical recommendations proposed by an international expert panel are designed (47–49). In order to rule out interference of results by antihypertensive drugs, the SPYRAL HTN-OFF MED (50) included patients with resistant hypertension who were not receiving antihypertensive medication or were able to tolerate discontinuation, and the SPYRAL HTN-ON MED (51) included the refractory hypertensive patients who were treated with three specific antihypertensive medications to standardize the antihypertensive drug regimen, and patients were instructed to take the prescribed antihypertensive medication before the start of each follow-up visit in order to eliminate interference of medication adherence with the results. In addition, the patients with isolated systolic hypertension (ISH) were excluded in the series trials of SPYRAL, and the hypertensive patients with relatively less comorbidities were included, reducing the heterogeneity of the study population (52). In terms of surgical operation, the SPYRAL multipolar catheter supported ablation in all four quadrants of the artery, and the main trunk, branches and accessory renal arteries were ablated, resulting in a wider range of ablation, furthermore, in terms of effect indicators, the change of ambulatory BP was the primary outcomes, leading to the more stable and reliable test results (52). The results of the two trials showed a significant decrease in BP from baseline in the RDN group at 3 or 6 months of follow-up, but not in the control group. The SPYRAL HTN-OFF MED Pivotal study (53), designed based on a novel Bayesian approach, validated its efficacy in a larger sample size of hypertensive patients, reported a further reduction in office BP in the RDN group compared with the control group after 3 months follow-up, mean difference was −6.6 mmHg (95% CI: −9.6 to −3.5, *p* < 0.0001).

The superiority results of the series studies have renewed interest in RDN and have led to the development of radiofrequency-independent ablation devices, such as ultrasound and chemical ablation-based RDN. The Paradise Catheter System (ReCor Medical, Palo Alto, CA, USA) is an ultrasound-based RDN device, the RADIANCE-HTN SOLO trial (54) confirmed the efficacy of the Paradise system in hypertensive patients not taking antihypertensive medication, with a further reduction in BP of −6.3 mmHg (95% CI: −9.4 to −3.1, *p* < 0.001) in RDN group compared with the control group after 3 months follow-up. The favorable outcome was also observed in 12 months follow-up (55). RADIANCE-HTN TRIO trial (8) validated the effectiveness of RDN when combined with antihypertensive drugs in patients with refractory hypertension. About 136 hypertensive patients whose BP remained poorly controlled after 4 weeks of treatment with a single-pill compound antihypertensive preparation were randomized to the RDN group vs. the sham-controlled group. After 2 months follow-up, the results showed that the RDN group was better than the control group in lowering BP [−8.0 vs. −3.0 mmHg, mean difference: −4.5 mmHg (95% CI: −8.5 to −0.3, *p* = 0.022)]. However, satisfactory results were not obtained in the recently published REQUIRE study (56), which included 143 Korean and Japanese patients with refractory hypertension who were randomly assigned to the RDN based on Paradise system and sham group on the basis of antihypertensive medications. There was no significant difference in 24-h SBP change between the RDN and sham groups at 3-month follow-up (−6.6 vs. −6.5 mmHg, *p* = 0.971). The authors claimed that various reasons should be considered for these negative results, including unstandardized antihypertensive regimens, unevaluated drug adherence, risk of study unblinding, and incomplete exclusion of patients with secondary hypertension. Rigorous RCT studies need to be designed to revalidate the antihypertensive effect of Paradise.

The Peregrine System (Ablative Solutions, San Jose, CA, USA) is an alcohol-mediated chemical ablation device that destroys perivascular nerves by injecting anhydrous alcohol through a catheter microneedle into the perivascular area of the kidney (57), offering a new option for RDN. Clinical studies on the feasibility of Peregrine have confirmed its effectiveness in lowering BP, with 45 patients with refractory hypertension treated with alcohol-mediated RDN having 24-h ambulatory systolic and diastolic BP decreases of 11 and 7 mmHg (all *p* < 0.001) from baseline to 6-month follow-up (58). The positive outcomes were also reported in 12 months follow-up and no procedure-related complications were observed (59). Effectiveness studies of Peregrine chemical ablation are underway to evaluate its efficacy and safety in the absence of antihypertensive medication (TARGET BP OFF-MED, NCT03503773) vs. in combination with antihypertensive medication (TARGET BP I, NCT02910414). In addition, some new RDN operating systems have been developed, such as the multipolar ablation catheter IberisBloom (Terumo, Tokyo, Japan) etc. (60–65) and the non-invasive stereotactic radiotherapy (66), whose clinical applications are expected.

Whether based on radiofrequency, ultrasound or chemical devices, RDN is effective in controlling BP levels in patients with resistant hypertension, but reliable head-to-head studies comparing the effectiveness between the approaches are lacking. Although the RADIOSOUND-HTN compared the Paradise and SPYRAL based RDN in lowering BP, and the results showed that ultrasound-based RDN lowered BP better than multipolar ablation catheter-based RDN (−13.2 vs. −6.5 mmHg, mean difference: −6.7 mmHg, *p* = 0.038), however, the study had a follow-up time of only 3 months and failed to effectively control for the interference of combined antihypertensive medications (67). Whether ultrasound-based RDN is superior to radiofrequency-based RDN needs to be confirmed by well-designed RCT studies.

### Arrythmia

Overactivation of SN plays an important role in the development and maintenance of atrial (68) or ventricular arrhythmias (VAs) (69, 70). Early clinical studies demonstrated that modulation of sympathetic tone was effective in controlling arrhythmias (71, 72). Pokushalov et al. (73) first demonstrated the value of RDN in atrial fibrillation (AF), 27 patients with drug-refractory AF were randomized to the pulmonary venous isolation (PVI) combined with RDN group and the PVI-only group, after 12 months of follow-up, the AF control rate of RDN group was significantly higher than the PVI-only group (69 vs. 29%, *p* = 0.033). The ERADICATE-AF study validated the effect in a larger paroxysmal AF population, evaluating the control of atrial arrhythmias in the PVI alone group (*n* = 148) vs. the PVI combined with RDN group (*n* = 154) at 12 months follow-up. The results showed that AF control rate was significantly higher in the RDN group compared with the PVI alone group (71.3 vs. 56.5%, *p* = 0.006) (9). It has been demonstrated that VAs can be effectively controlled by regulating sympathetic activity (74–76). Ukena et al. (77) firstly demonstrated the efficacy of RDN in the control of VAs in two patients with hypertrophic cardiomyopathy combined with sympathetic electrical storm who had a significant reduction in VAs after RDN. An international multicenter clinical study that included 13 patients with refractory VAs confirmed that the frequency of ventricular tachycardia/ventricular fibrillation episodes decreased from 21 times per month to 2 times per month (*p* = 0.004) at 1 month after RDN (78), and a recent retrospective study also confirmed the efficacy of RDN on VAs after cardiac denervation (79). Due to enrollment difficulties, there is a lack of RCT studies with large samples to confirm the role of RDN in controlling VAs.

The mechanisms of RDN in anti-arrhythmia are not fully understood, and although the ERADICATE-AF study (9) confirmed that RDN was effective in controlling AF recurrence, the study did not elucidate whether this result was related to altered sympathetic activity, in other words, the effect may be related to the underlying mechanism of RDN, but not to changes in sympathetic activity.

### Heart Failure

Sympathetic overactivation is an important pathophysiological feature of HF (80), and the release of norepinephrine after SN activation is an important predictor of poor prognosis in HF patients (81). Few studies have been conducted on RDN for HF, and the REACH Pilot study was the first to confirm the efficacy of RDN in heart failure with reduced ejection fraction (HFrEF), the study included seven patients with HFrEF receiving standardized pharmacological therapy, 6 months after receiving RDN, the 6-min walking distance (6MWD) increased significantly from baseline (mean difference: 27.1 ± 9.7 m, *p* = 0.03) (82). The efficacy of RDN for HFrEF was further confirmed in subsequent clinical studies with larger sample sizes, with several studies showing that RDN could improve ejection fraction (EF), New York Heart Association (NYHA) cardiac function class, and reduced brain natriuretic peptide (BNP) levels (10, 83, 84). A meta-analysis of 5 clinical studies with 177 patients showed that RDN increased EF in patients with HFrEF (weighted mean difference: 6.289%, 95% CI: 1.883–10.695%) (85), providing a theoretical basis for a large RCT study. In addition to HFrEF, many heart failure patients present with preserved ejection fraction (HFpEF), and the pathophysiological mechanisms of HFpEF are also associated with increased sympathetic tone, but the available evidence does not establish a causal relationship between them (86). Few studies have been conducted on RDN in patients with HFpEF, and no consistent findings have been reached in previous animal studies (87, 88). Two related clinical studies (DIASTOLE, NCT01583881, RDT-PEF, NCT01840059) had to be pre-maturely discontinued because of enrollment difficulties, although it was not possible to systematically assess the efficacy of RDN in HFpEF patients, published results from the RDT-PEF study (89) showed that RDN improved ventricular diastolic function in HFpEF patients. Results of a multicenter CMR imaging study also showed that RDN improved ventricular longitudinal strain and thus ventricular diastolic function in 16 patients with resistant hypertension combined with HFpEF (90).

Conventional anti-HF drugs (β-blockers, ACEI/ARB, etc.) put effect on the intermediate conduction phase of neurohumoral regulation or target organ receptors, thereby inhibiting sympathetic activity, but these approaches fail to address the upstream enabling segments that promote the progression of HF. RDN also inhibits afferent signals transmitted to the CNS, efferent signals transmitted to the kidney and other target organs, and related downstream signaling pathways (β1-adrenergic receptors, the RAAS system, and enkephalinase), thus improving HF prognosis more than related drugs (36). We expect future large-scale clinical studies to confirm this hypothesis.

### Other Applications

In addition to CVDs, a small number of studies have reported the use of RDN in other systems. RDN may protect the kidneys through mechanisms such as lowering BP and inhabiting the renal inflammatory response (91, 92). Studies have shown that RDN is useful in relieving obstructive sleep apnea (93), lowering blood glucose (94), and blood lipids (95), improving pulmonary hypertension and inhibiting pulmonary artery mechanical remodeling (96) and depressed mental status (97). Various factors such as single-center, open-label, and small samples, however, could affect the authenticity of trial results, and we urgently need rigorous and standardized RCT studies to confirm the effects of RDN.

### Summary and Future Outlook

Based on the theory and data, we believe that RDN is a novel and effective non-pharmacological means of controlling hypertension, although the mechanism is not fully defined. After excluding the secondary hypertension, patients with uncontrolled hypertension despite guideline-based antihypertensive therapy, poor medication compliance, high risk of cardiovascular events or existing cardiovascular events and target organ damage may be the best candidates for RDN. In patients with ISH, RDN is still not recommended by current expert consensus due to limited clinical evidence (98). Although some studies have reported the effects of RDN in non-CVDs, they are not recommended for use in relevant patients because the studies are in the exploratory phase and the efficacy and safety are unclear.

RDN is a new device-based therapy for regulating sympathetic tone, and many questions remain unanswered:

(1) Long-term efficacy and safety are unclear: RDN can only destroy nerve fibers around the renal artery and cannot affect the nerve body upstream of it, therefore, the possibility of reinnervation exists. Booth et al. (99) has demonstrated that significant sympathetic nerve functional and anatomical reinnervation had occurred by 5.5 months after RDN in sheep. Accordingly, clinicians should pay more attention to the long-term hypotensive effectiveness. The most published RDN studies followed-up within 12 months, and although the 3-year follow-up results of the first-generation ablation catheter SYMLICITY suggested good efficacy and safety of RDN (100), the long-term efficacy and safety of new generation ablation catheters such as Peregrine and Paradise are unclear.
(2) The standard procedure remains controversial: There are still debates about whether to ablate the whole segment of the main trunk of the renal artery bilaterally or to ablate the main trunk of the renal artery combined with the branches, although anatomical studies have confirmed that a considerable number of sympathetic nerve fibers of the renal artery cross the main trunk to directly innervate the branches (23). However, Fengler et al. suggested that ablation of the main renal artery alone was superior to combined branch ablation (67).
(3) The ablation endpoints are elusive: Since RDN has been performed, there have been no clear ablation endpoints, and operators are often unable to judge the success of the procedure. Although renal SN stimulation may help to determine the effect of RDN (101), it may be too complicated and time-consuming to be clinically useful. Future efforts should focus on exploring a safe and reliable method of perioperative evaluation.
(4) The cardiovascular benefits are unclear: Although the reduction of BP in hypertension can bring cardiovascular benefit theoretically (102), and a secondary study using multiple regression models hypothesized that RDN can significantly reduce the risk of cardiovascular events by lowering BP (103), no clinical study data are available to confirm the clinical benefit of RDN (104). The issues above need to be confirmed by rigorously designed large-scale RCT studies.

## Pulmonary Artery Denervation

### Anatomy and Mechanism

Numerous animal studies have demonstrated that pulmonary SNs supplying the pulmonary vascular tree origin from the first five thoracic ganglia, the stellate ganglia, and the middle and inferior cervical ganglia, and the sympathetic post-ganglionic fibers from these ganglia enter the lung and innervate the pulmonary vessels (105). It is noteworthy, however, that the fat and connective tissue surrounding the pulmonary artery trunk is also rich in sympathetic fibers (106), which are adjacent to the cardiac autonomic nerve and the right phrenic nerve, and that treatment with PADN may cause damage to these adjacent nerves. However, most of the above anatomical evidence is derived from animal studies, and there are significant differences in pulmonary nerve distribution between species (107). There is less anatomical evidence on the innervation of the pulmonary arteries in humans. Therefore, we need large samples of human autopsy evidence to support the rationale for performing PADN. Rothman et al. (108) performed two human autopsies (one male and one female) and demonstrated by immunohistochemical techniques with tyrosine hydroxylase staining that the nerve fibers surrounding the pulmonary arteries were predominantly sympathetic (71% of fibers stained positive), and that these nerve fibers were mainly distributed in the right and left main pulmonary arteries with a thickness >4 mm.

Over the past few decades, a large body of research evidence has supported an important role for sympathetic nerves in the development of pulmonary hypertension (PH). As early as 1952, De Burgh Daly et al. (109) demonstrated in animal studies that SN stimulation increased pulmonary vascular resistance (PVR) at constant blood flow, and subsequent studies further demonstrated that sympathetic overexcitement decreased pulmonary vascular compliance and increased pulmonary circulatory resistance, thereby increasing pulmonary artery pressure (PAP) (110). Velez-Roa et al. (111) compared muscle sympathetic nerve activity (MSNA) in PH patients with that in healthy subjects by microneurography and showed that MSNA was significantly higher in the PH group, providing the first direct test of the hypothesis that SN activity was increased in patients with PH. In addition to directly increasing PVR, sympathetic hyperactivation may also promote pulmonary vasoconstriction, endothelial cell proliferation and migration, extracellular matrix remodeling, and cellular fibrosis through modulation of RAAS system activity, leading to pulmonary vascular remodeling and pulmonary arterial hypertension (112). A study analyzed blood specimens from 79 patients with idiopathic pulmonary arterial hypertension (IPAH) and showed significantly elevated levels of renin, angiotensin I, and angiotensin II (113), further suggesting a role for RAAS activation in PH. Although the specific mechanisms of SN involvement in the pathogenesis of PH are not fully understood, the above studies have suggested that modulation of pulmonary sympathetic tone may be effective in the control of PH.

### Clinical Evidence

Although the surgical PADN technique has been reported in 1980s (114), it requires general anesthesia and transthoracic surgery, which is intolerable for most patients with PH. In 2013, Chen et al. in China first proposed a new method to treat PH using transcatheter-based pulmonary artery denervation with good results (115). In this single-center, open-label clinical study, 21 patients with refractory IPAH were divided into the PADN group and the drug treatment group according to the patients' wishes, and after 3 months of follow-up, there was a significant decrease in mean pulmonary artery pressure (mPAP) (55–36 mmHg, *p* < 0.01) and a significant improvement in 6 WMT (324–491 m, *p* < 0.006) in the PADN group, whereas there was no significant change in the control group. However, this study has been widely controversial due to the limitations of the small sample, open-label and short follow-up time. A subsequent study by Chen et al. confirmed the feasibility of PADN in a larger sample size population (116), 66 patients with different types of PH were all treated with PADN, and there was a significant decrease in mPAP, right atrial pressure and PVR after 1 year (all *p* < 0.05). In fact, the most significant decrease in mPAP was observed at 6 months, with no further decrease at 12 months. In terms of safety, the incidence of PH-related adverse events (AEs) at 12 months follow-up was 15%, which is lower than that of the classical drug regimen (117), further confirming the efficacy and safety of PADN. Although the above studies provided preliminary evidence that PADN may be effective in reducing PAP, they were studies of open-label, non-randomized, and without sham-operative controls. To exclude the above bias from interfering with the study results, Chen et al. designed a multicenter, randomized, sham-controlled study (118), aiming to verify the value of PADN in patients with pre-capillary combined with post-capillary, the results showed that PADN was effective in reducing PAP and improving 6 WMT levels in patients with no increase in procedure-related AEs compared with control group.

In addition to the electrode ablation device proposed by Chen et al., several new PADN devices have been available. Romanov et al. (119) proposed a new PADN device based on the remote magnetic navigation system (Niobe ES, Stereotaxis, St. Louis, Missouri) which can automatically locate the ablation catheter. Combining it with a three-dimensional electrophysiological landmarking system (CARTO-RMT, Biosense Webster, Inc., Diamond Bar, California) could facilitate radiation reduction and precise ablation, in this study, 50 patients with chronic thromboembolic pulmonary hypertension whose PAPs were still uncontrolled after pulmonary artery endarterectomy and 6 months of continuous optimal drug therapy were randomly divided into PADN and sham-procedure group, the results showed that mPAP, PVR, and 6 MWT were significantly improved in the PADN group compared with the control group, and no procedure-related serious AEs were observed. The TIVUS system is an intravascular ultrasound-mediated PADN device that produces a thermal area of effect at a depth of ~10 mm, and its effectiveness in mediating PADN has been demonstrated in pre-clinical study (108). The TROPHY I (120), a clinical study on the feasibility of the TIVUS system, included 23 patients with PH who did not respond to vasodilators were treated with PADN, the incidence of procedure-related AEs and changes in hemodynamic parameters of the pulmonary circulation were observed at 4–6 month follow-up, and the results showed that no AEs occurred during the follow-up period. There was no significant change in mean pulmonary artery pressure in the immediate post-operative period, which was different from the decrease in mPAP in the immediate post-operative period observed by Chen et al. (115), but at 4–6 month follow-up, PAP, PVR and 6 MWT were significantly improved (all *p* < 0.05). The ongoing TROPHY II study (NCT03611270) will validate the safety and preliminary effectiveness of the TIVUS System in patients with PH associated with left heart disease. The TROPHY PAH Pivotal (NCT04570228) is a prospective, multicenter, randomized, sham-controlled study that will validate the safety and efficacy in a larger sample size. The nMARQ loop ablation catheter (Biosense Webster Inc., Diamond Bar, CA) is another multi-electrode ablation device for which a feasibility study for PADN application (NCT02403908) is underway. Recently, an animal trial reported the use of laser energy for PADN and confirmed sympathetic nerve destruction in the pulmonary artery on histopathological examination, providing a new idea for PADN (121). In addition to the above regulation modalities, study has also reported the use of chemical drugs for PADN (122), but the currently used 6-hydroxydopamine can lead to cardiac autonomic dysregulation and myocardial injury (122), and is difficult to use in clinical practice.

The PADN technique is still in the validation phase, and relevant clinical studies are limited and restricted to patients with PH. Although some studies have reported that PADN can affect cardiac hemodynamics (118), the precise mechanisms of PADN are unclear, and further studies are needed to confirm whether PADN can provide a benefit in non-pulmonary vascular diseases.

### Summary and Future Outlook

PADN is a very novel non-pharmacologic therapy for PH, providing a new treatment option for patients with drug-refractory PH, but it also raises some new issues and challenges:

(1) The optimal target population is unknown: According to the pathophysiologic mechanism, PH can be broadly divided into pre-capillary and post-capillary PH. Post-capillary PH, also known as left heart disease-associated PH (LHD-PH) is the most common type of PH in clinical practice (123), but the current common pharmacological regimen for PH is not recommended for LHD-PH because most trials have proven to be ineffective or harmful (124). The underlying factors in the formation of LHD-PH are elevated left ventricular end-diastolic pressure and functional mitral regurgitation, which may not be affected by PADN (125). The results of a small sample of clinical studies by Chen et al. suggest that PADN may be beneficial in patients with LHD-PH (116, 118), but well-designed clinical studies are still needed to confirm this view.
(2) The standard ablation procedure is unavailable: Current ablation procedure mostly follows the distal pulmonary artery trunk and the whole proximal segment of the right and left pulmonary arteries as described by Chen et al. (115), but this extended ablation approach is more prone to adverse events such as pulmonary stenosis and fibrosis. The anatomical background of the sympathetic innervation region of the pulmonary arteries in humans is poorly understood and the exact distribution of the sympathetic nerves and the location of ablation cannot be clarified. However, Goncharova et al. (126) recently proposed to use the response to intrapulmonary artery high-frequency electrical stimulation, such as heart rate changes, coughing and eructation, to precisely mark the ablation site while avoiding damage to the adjacent phrenic or recurrent laryngeal nerves, providing a new perspective for selective PADN. In addition, nuclear medicine cardiac imaging techniques have been used for precisely mapping of cardiac autonomic ganglionated plexi (GP) then to guide GP ablation (127), and similarly, may be used for precise localization of pulmonary sympathetic nerves to guide selective PADN.
(3) The ablation endpoints are controversial: Although the study by Chen et al. (115) claimed that a decrease in mPAP measured by right heart catheterization in the immediate post-operative period compared with the pre-operative period of ≥10 mmHg, this number may be empirical and not supported by relevant studies, and this criterion may be variable for patients with different degrees of PH. Ablation endpoints may be various for different ablation devices, and a decrease in mPAP in the immediate post-operative period was not observed in the study of ultrasound-guided PADN (120), therefore, we need a safe and reliable way for perioperative evaluation.
(4) The long-term efficacy and safety are unclear: As mentioned above, PADN is a novel treatment for PH, and limited clinical studies have been conducted, the long-term efficacy and safety issues of PADN have not been validated, which determines whether PADN has value in clinical application. In addition, although the reduction of PAP in PH patients is accompanied by clinical benefit (124), no studies have been conducted to confirm the specific benefit of PADN after PAP reduction. These questions and challenges may be the key directions for future research in the field of PADN.

## Hepatic Artery Denervation

### Anatomy and Mechanism

The sympathetic splanchnic nerves innervating the liver originate from neurons in the celiac and superior mesenteric ganglia, which are innervated by pre-ganglionic neurons located in the intermediolateral column of the spinal cord (T7–T12) (128). The perihepatic plexus can be roughly divided into an anterior plexus, which runs with the hepatic artery, and a posterior plexus, which runs along the portal vein and bile duct. They give out two clusters of SN in the hilar region that enter the liver with the hepatic artery and portal vein, with some SNs extending to the pancreas and duodenum (129). There is the evidence from anatomical studies that the vast majority of nerve fibers around the common hepatic artery are sympathetic rather than parasympathetic (130), which provides an anatomical basis for the HADN *via* the common hepatic artery.

Liver is an important organ involved in metabolic regulation, which depends heavily on the autonomic nerves action. Autonomic modulation of the hypothalamus was shown to control glycogen metabolism (131, 132). Viral reverse tracing techniques have further demonstrated a direct link between hypothalamic and hepatic innervation (133), with stimulation of sympathetic efferent nerves leading to increased hepatic glucose output through activation of glycogen phosphorylase and increased phosphoenolpyruvate carboxykinase activity, resulting in the increase of blood glucose concentration (134). In addition to its involvement in glycogen metabolism, liver also plays a key role in regulating lipid metabolism. Hepatic sympathetic hyperexcitability can facilitate the release of neuropeptide Y, which promotes the maturation and release of very low density lipoproteins, leading to the development of hyperlipidemia (135). Liver has critical roles in glucolipid metabolism, cellular immunity, and stem cell remodeling and regeneration, but the specific mechanisms need to be confirmed by further basic studies.

### Clinical Evidence

The vast majority of HADN is currently performed by surgery, mainly by dissection of nerve fibers from the celiac ganglion plexus 1–2 cm away from the beginning of the common hepatic artery to a few centimeters after the bifurcation of the common hepatic artery (136). Results of previous animal trials have confirmed the function of surgical HADN for improving metabolic diseases such as diabetes (130, 137, 138), dyslipidemia and hepatocellular steatosis (139–141). However, surgical HADN is hardly applicable to clinical treatment because it requires general anesthesia and laparotomy, which is more invasive. Due to the intensive research on RDN, people no longer limit their attention to the interventional modulation of renal SN, and propose using catheter ablation for HADN to avoid the huge invasiveness of surgical procedures. In 2014, Webster's team in New Zealand proposed to use a novel ablation device, the Metabolic Neuromodulation System (Metavention, Maple Grove, MN, USA) to perform catheter ablation of the common hepatic artery to modulate hepatic SN activity, and conducted the first-in-human clinical trial of catheter-based HADN, the COMPLEMENT study (NCT02278068). The study aimed to evaluate safety of the device in type 2 diabetes patients with poorly controlled glucose by current medications and its effect on glycemic and others. The results of the study are expected to be published shortly. In addition, the Metavention has developed an integrated catheter ablation system, the iRF Denervation System (Metavention, Maple Grove, MN, USA), which allows for combined denervation of the hepatic and renal arteries. A significant decrease in norepinephrine concentrations in liver and kidney tissues at 90 days after the procedure compared with baseline (all *p* < 0.05), suggesting the possible effectiveness of combined ablation (142). Clinical studies on the iRF system were also conducted in 2020, and the DeLIVER study (NCT04285554) aimed to explore the safety of the iRF system and its effects on metabolic markers such as blood glucose and lipids, and its results are expected. Although there are few clinical studies on transcatheter HADN, the success of the RDN and PADN series trials gives us hope for a new approach to transcatheter HADN for the regulation of metabolic process.

### Summary and Future Outlook

Liver plays a key role in the metabolic processes such as glycogen synthesis and lipid transport and degradation, and there are dense autonomic nerve fibers in the perihepatic and intrahepatic regions, the metabolic processes can be regulated by modulating hepatic autonomic nerve activity. HADN provides a minimally invasive way to regulate hepatic autonomic nerves, offering a new therapeutic idea for patients with drug-refractory metabolic diseases such as diabetes and hyperlipidemia. Transcatheter HADN is a novel therapeutic tool and its clinical feasibility is unknown as the results of the first-in-man study have not yet been published, but the results of previous animal studies have shown that HADN is effective for metabolic modulation of blood glucose and lipids. We expect that the publication of the clinical results will provide new therapeutic options for patients with drug-refractory metabolic diseases. It is noteworthy that the SN fibers in the perihepatic regions also send out branches to innervate the pancreas, duodenum and other organs (129), and it is likely that radiofrequency energy destroys the perihepatic nerve fibers and damages these branches as well, and to put effect on the functions of these organs.

In addition, the advent of iRF system has raised our interest in multi-organ denervation (MDN). It is known for us that SN is widely distributed in internal organs such as the heart, lung, and kidney, and that localized denervation of a single organ may not completely suppress the over-activated sympathetic nervous system activity. The constantly updating of denervation devices has opened our eyes to the possibility of MDN in the future, which may theoretically regulate sympathetic activity at a greater degree, therefore, may lead to greater clinical benefit. This may serve as a key research direction in the field of device-based regulation of automatic nervous system in the future.

## Splenic Neuromodulation

### Anatomy and Mechanism

The nerves that innervate the spleen are mainly sympathetic post-ganglionic fibers originating from the celiac ganglion and superior mesenteric ganglion, which enter the spleen along with the splenic artery, and then follow the central splenic artery to the splenic white medulla and eventually to the periarterial lymphatic sheath (143, 144). In addition to the vast majority of sympathetic fibers, it has been found that there may be parasympathetic innervation of the splenic apical region (145). The spleen is the largest secondary lymphoid organ and has a wide range of immunomodulatory functions, with a key role in both cellular and humoral immunity (146). Specifically, the spleen is involved in antigen presentation, T-cell activation, and B-cell differentiation into antibody-producing splenic plasma cells, and is an important organ in the adaptive immune response of the body (147). When the splenic SNs are destroyed, antigen-induced antibody secretion by plasma cells is significantly reduced (148). It has been found that splenic SN can also regulate splenic blood flow by controlling splenic artery contraction (149). Recently, scholars have focused on the overactivation of the immune system as an important factor involved in the pathogenesis of hypertension. Although the exact molecular mechanisms are not fully understood, it has been demonstrated that immune-mediated oxidative stress can trigger interstitial inflammation and peritubular capillary injury in the kidney, resulting in decreased water and sodium excretion, which in turn causes an increase in BP (150). Carnevale et al. (151) suggested that stimulation of splenic nerve could induce a transient increase in BP and that SADN could effectively lower BP, this effect was related to the inhibition of T-cell activation, migration and consequent infiltration of target organs by SADN. This undoubtedly provides a new treatment idea for resistant hypertension.

The role of the splenic autonomic nervous system in the regulation of the inflammatory response is still controversial. Previous studies have shown that splenic sympathetic activation exerts an anti-inflammatory effect (152). After the central nervous system senses the over-activated inflammatory response of the organism, it generates action potentials, which are transmitted to the ventral ganglion through the cholinergic fibers of the vagus nerve, that in turn activates the splenic sympathetic nerve to release norepinephrine. Norepinephrine binds to the β2-adrenergic receptors of splenic T lymphocytes to release acetylcholine, and finally acetylcholine acts on the α7 nicotinic acetylcholine receptors on the macrophages of the splenic red marrow and the marginal zone, which could depress the release and aggregation of inflammatory factors such as TNF-α (153). This pathway is now known as the inflammatory reflex (154–156). Subsequent animal experiments have shown that the concentration of pro-inflammatory factors such as IL-1β and IL-6 increased significantly after destruction of the splenic nerve (157), which further demonstrated the anti-inflammatory effect of the splenic nerve activation. Kressel et al. (158) reported that transcutaneous auricular vagus nerve stimulation could suppress immune system and inflammatory response, the stimulation signal can be transmitted through cholinergic neurons in the brainstem dorsal motor nucleus of the vagus projecting to the celiac-superior mesenteric ganglia and transmitting cytokine-inhibiting signals to the splenic nerve. However, previous studies have generally concluded that the splenic nerve is adrenergic rather than cholinergic and cannot explain how the α7nAChR in the spleen senses signals from cholinergic nerve fibers. With further studies, it has been found that the autonomic nervous system of the spleen also contains a small number of cholinergic nerve fibers innervating the splenic apical region (145, 159), which may be an important approach in the involvement of splenic autonomic nerves in the regulation of inflammatory responses.

Although it has been shown that stimulating splenic sympathetic nerve helps to suppress the inflammatory response, the results of Albaghdadi et al. (160) showed that SADN could reduce the levels of inflammatory factors such as TNF-α, IL-1β, and IL-6 in a porcine model of rheumatoid arthritis. There is a contradiction between the above findings, and the inflammatory regulatory effects of splenic nerve activation and splenic denervation need to be studied in depth.

### Clinical Evidence

Studies on splenic neuromodulation have been conducted on animal models, and no studies have reported the application of splenic neuromodulation in human. Although Albaghdadi et al. (160) initially demonstrated the feasibility of modulating splenic nerve activity by catheter ablation using the NEXION Autoimmune Modulation System (LaVita Technologies, Mount Laurel, NJ, USA), the results of their study warrant further discussion. The Galvani System (Galvani Bioelectronics, South San Francisco, CA, USA) is an implantable splenic nerve modulation device in which stimulation electrodes are surgically implanted around the splenic artery and a pulse-emitting device is implanted under the skin to artificially regulate splenic nerve activity, as demonstrated in preliminary animal studies, intermittent stimulation of the splenic nerve is effective in reducing the level of endotoxin-induced inflammatory factors (161). Two feasibility studies (NCT05003310, NCT04955899) are underway on the Galvani System for the treatment of rheumatoid arthritis, which will be the first application of splenic nerve device regulation in humans, and the results of which are expected.

### Summary and Future Outlook

Spleen is an important immune organ, and regulation of splenic nerve activity can effectively modulate systemic inflammatory and immune responses. However, the specific effects of splenic nerve modulation are not fully understood and should be the focus of future investigation. Inflammation and immune responses are involved in the pathogenesis of various diseases such as atherosclerosis, myocardial infarction, and myocarditis (162), regulating the inflammatory and immune processes to treat relevant diseases has promising applications. For example, fulminant myocarditis is an acute CVD with a high mortality rate, and there is a lack of effective treatment modalities. Over-activation of myocardial inflammation and immune response is the main mechanism of its rapid progression (163), inhibition of excessive myocardial inflammatory and immune responses using splenic neuromodulation may provide a new therapeutic option.

## Other Regulation Methods

### Baroreflex Activation Therapy

Baroreceptors located in the carotid sinus and the aortic arch regulate inappropriate BP changes in real time by modulating the tone of the autonomic nervous system (164). When BP increase, arterial wall tone increases accordingly, which in turn activates the baroreceptors, causing a decrease in sympathetic efferent signals and a decrease in total peripheral resistance of the body, exerting a lowering effect on BP (165). In the 1960s, Rothfeld et al. (166) reported a successful case of refractory hypertension treated with baroreflex activation therapy (BAT), which was the first use in humans. The Rheos system (CVRx, Inc., Minneapolis, Minnesota), a first-generation carotid sinus electrical stimulation device, was similar to a pacemaker in that electrodes were placed around the carotid sinuses bilaterally and stimulation signals were delivered using a subcutaneously implanted pulse transmitter. The previous research demonstrated the efficacy in resistant hypertension (167–169), and the reduction in BP was closely associated with a decrease in heart rate, muscle sympathetic activity, and plasma renin concentration (170, 171), confirming that this hypotensive effect was achieved through inhibition of sympathetic activity. Although the first-generation Rheos system was effective in lowering BP, it suffered from common procedure-related complications such as facial nerve injury and the need for frequent battery changes, and its indication for lowering BP was not approved by the FDA (172). The Barostim Neo system (CVRx, Inc., Minneapolis, Minnesota), a second-generation carotid sinus electrical stimulation device, overcomes these disadvantages, has a longer battery life, requires unilateral carotid sinus peri-implantation of the stimulating electrodes, and has been shown to be effective in controlling hypertension in several clinical studies (173–175). Although the system has a high rate of AEs, with a small sample study showing mild local adverse effects in 97.6% of subjects, most of which can be resolved by adjusting the pulse transmitter settings (176). In addition to lowering BP, the BeAT-HF study confirmed that BAT with the Neo system was beneficial in patients with HFrEF. The HFrEF patients were randomized to Neo treatment group combined with a drug treatment group and drug treatment alone group. The 6 months follow-up results showed that compared with the drug treatment group, patients in the BAT treatment group had a 60 m increase in 6 MWT (95% CI: 40–80, *p* < 0.001), and there was also a significant decrease in NT-proBNP (Δ = −25%, 95% CI: −38 to −9%, *p* = 0.004) (177).

Although carotid sinus electrical stimulation therapy may be effective and relatively safe in patients with refractory hypertension, the method is invasive and requires surgical placement of electrodes around the carotid artery. Moreover, the antihypertensive effects of the second-generation Neo system are not yet supported by RCT results, and we look forward to the results of the Nordic BAT study (NCT02572024), a randomized controlled trial. Carotid sinus electrical stimulation has a unique advantage over other sympathetic device modulation devices in that immediate BP changes can be observed by turning the pulse transmitter on and off, which can help identify positive responders.

### Endovascular Baroreflex Amplification

Endovascular baroreflex amplification (EBA) is similar to BAT in that both regulate sympathetic activity by modulating baroreceptor reflexes in the carotid sinus and aortic body. Unlike the electrical stimulation modulation of BAT, EBA continuously activates the pressure reflex by producing a sustained pull on the vessel wall through implantation of a self-expanding stent in the carotid sinus region (164). The CALM-FIM_EUR (178) was the first-in-man study to validate the efficacy of EBA, which included 30 patients with refractory hypertension who received implantation of the EBA device MobiusHD (Vascular Dynamics, Mountain View, CA, USA). There was a significant decrease in office BP and 24-h ambulatory mean BP from baseline at 6-month follow-up (all *p* < 0.05) with the satisfactory safety. The multicenter, randomized, double-blind studies, CALM-START (NCT02804087) and CALM-2 (NCT03179800), will further confirm the efficacy and safety of EBA, and their results are promising. Since EBA requires metal stent implantation, its post-operative antithrombotic regimen and the possible bleeding events caused by these drugs require additional attention. In addition, cerebrovascular complications such as contrast encephalopathy, thromboembolism and carotid plaque dislodgement that may be caused by carotid angiography and carotid stenting need to be closely monitored in the perioperative period.

### Transvenous Carotid Body Ablation

The carotid body, located at the bifurcation of the carotid artery, is the main peripheral chemoreceptor in humans, and enhanced carotid body-mediated chemoreflexes can cause increased sympathetic activity. Although the exact mechanisms of action are not clear, modulating carotid body activity may have some influence on BP regulation (179–181). Narkiewicz et al. (182) first described the function of regulating carotid body activity in patients with hypertension, fifteen patients with refractory hypertension underwent unilateral carotid body surgical resection, during the 12 months follow-up, no significant changes in BP from baseline were found in the subjects, however, eight patients had a significant decrease in 24-h ambulatory systolic BP at 3 months follow-up. The study also reported nine AEs associated with the procedure, including surgical wound hematoma and infection. The study published in 2017 reported a first-in-man study of transvenous carotid body ablation (TCBA) in resistant hypertension (183), which used the Cibiem Transvenous Ultrasound System (CTUS) (Cibiem, Inc., Los Altos, CA, USA) to perform transcatheter-based ultrasound ablation of the carotid body *via* internal jugular vein, the 6 months follow-up of 15 patients with refractory hypertension treated with TCBA showed a 9 ± 9 mmHg and 4 ± 6 mmHg reduction in 24-h ambulatory systolic and diastolic BP from baseline, with no serious AEs occurred during the follow-up period. Another study published in 2018 confirmed the efficacy and safety in the relatively large population, 27 refractory hypertension patients received TCBA with a reduction in 24-h ambulatory systolic BP of 9.1 ± 13.5 mmHg vs. 4 ± 6 mmHg at 6 months follow-up compared with baseline, and 7 serious AEs were recorded, including transient ischemic attacks, hypotension and chest pain (184).

Although surgical excision of the carotid body does not significantly reduce BP, TCBA provides an interventional means of regulating carotid body activity, and it is unclear why the difference in efficacy between the two is significant. Previous small sample studies have reported good results with TCAB for hypertension (183, 184), and we look forward to the formal publication of the results (NCT03314012).

### Spinal Cord Stimulation

Spinal cord stimulation (SCS) mostly involves placing stimulating electrodes in the posterior part of the epidural cavity of the patient's spinal canal and stimulating the nerve conduction bundles in the posterior column of the spinal cord by adjusting the intensity of the stimulating current, which is mostly used for pain relief (185). With further research, it is now believed that SCS is effective in relieving myocardial ischemia and HF in several ways (186), and the mechanisms may be related to the inhibition of sympathetic signal efference (187, 188). Stimulation of the spinal nerve in the corresponding segment by a pulse transmitter can block the pain conduction pathway, and Ekre et al. (189) demonstrated that SCS can effectively relieve symptoms and improve quality of life in patients with refractory angina pectoris. The value of SCS in the treatment of HF was firstly demonstrated by Tse et al. (190), in a study of 17 patients with HFrEF who underwent high thoracic segment SCS combined with optimal drug therapy, after 6 months follow-up, the results showed the significant improvements in Minnesota Heart Failure Questionnaire score (42 vs. 27, *p* = 0.026), NYHA classification (3.0 vs. 2.1, *p* = 0.002), and LVEF (25 vs. 37%, *p* < 0.001), and no device-related serious AEs were identified. However, the subsequent DEFEAT-HF study (191) reported the opposite results, after 6 months follow-up, the left ventricular end-systolic volume index (LVESVi) was not significantly improved in the SCS group compared with the control group (2.1 vs. −2.2, *p* = 0.30), which stalled the study of SCS in HF. By regulating spinal sympathetic efferent activity, it may help to control some arrhythmias, and previous results in animals have shown that SCS significantly reduces the incidence of VAs after myocardial infarction (192–194). However, the use experiences of SCS in human are insufficient, Grimaldi et al. (195) reported 2 cases of VAs treated with SCS, showing that SCS significantly reduced the incidence of VAs, which provided a basis for future clinical studies. The ongoing TerminationAF study (NCT03539354) aims to explore the value of SCS in patients with AF, and its results are expected.

### Indirect Regulation Methods

Unlike the sympathetic regulation methods described above, indirect regulation methods require the placement of “receptors” inside or on the surface of the body that can sense specific external stimulus, and then the use of external energy such as electromagnetic fields (EMF) to signal to the “receptors,” and the “receptors” then exert their effects on neural activity. Transcranial EMF stimulation has been used to treat a variety of neurological disorders such as depression (196), Wang et al. (197) found that intermittent low-frequency EMF stimulation of the left stellate ganglion (LSG) significantly reduced the incidence of VAs after myocardial ischemia in an animal model, and the first-in-man application of low-frequency EMF stimulation was described in a subsequent series of case reports (198), which included 5 patients with sympathetic electrical storms who had failed to respond to pharmacological treatment, the electromagnetic coil was placed on the surface of the seventh cervical spine near the LSG, and intermittent low-frequency EMF stimulation was administered, showing a significant reduction in the incidence of VAs and no device-related AEs. The STAR-VT study (NCT04043312) on low-frequency EMF stimulation for sympathetic electrical storm is ongoing. In addition, low-intensity focused ultrasound stimulation of the LSG has also been suggested to reduce the incidence of infarct-related VAs (199).

Nanomedicine, an interdisciplinary approach combining modern medicine and nanotechnology, has been used for cardiac autonomic regulation in recent years. Yu et al. (200) found that magnetic nanoparticles carrying neurotoxic drugs were injected into the coronary arteries of dogs, under the positioning of an external magnetic field, the magnetic nanoparticles could precisely target the cardiac autonomic GP and inhibit its function, thus exerting an anti-arrythmia function. This technique provides a new idea for the prevention and treatment of cardiac arrhythmias, although sympathetic modulation of nanomedicine has not been reported yet.

## Conclusion

A variety of device-based SN modulation equipments could function through different pathways to lower BP, control arrhythmias and benefit HF (Table 1). Although the precise mechanisms are still unclear, a large number of clinical studies have confirmed the clinical application value of device-based SN modulation, which has a broad application prospect. On the other hand, because the clinical evidences are not sufficient, the long-term effectiveness and safety of most device-based SN modulation techniques are unknown, and their optimal modulation approaches, evaluation indicators, modulation parameters and potency ratio need to be confirmed by large-scale clinical studies. In addition, device-based SN regulation is invasive to some extent, which is one of the main factors limiting their widespread application. Based on the above, we believe that device-based SN regulation offers new therapeutic options for some CVDs, but drugs remain the preferred treatment option at present because the evidence for clinical application is not yet sufficient.

**Table 1:** Results of the device-based sympathetic nerve regulation in cardiovascular diseases.

**Trial**	**Year**	**Device**	**Follow up**	**No. in EG**.	**No. in control**	**Population**	**Comparison**	**Primary end point**	**Primary outcome (EG.)**	**Primary outcome (control)**	***P-*value**
**RDN**											
SYMPLICITY HTN-3	2014	Symplicity flex	6M	364	171	RHTN	RDN vs. sham	OSBP change	−14.13 ± 22.93 mmHg	−11.74 ± 25.94 mmHg	0.26
SPYRAL HTN-OFF MED	2017	Symplicity spyral	3M	38	42	RHTN	RDN vs. sham	24-h SBP change	−5.5 (−9.1 to −2.0) mmHg	−0.5 (−3.9 to 2.9) mmHg	0.0414
SPYRAL HTN-ON MED	2018	Symplicity spyral	6M	38	42	RHTN	RDN vs. sham	24-h SBP change	−9.0 ± 11.0 mmHg	−1.6 ± 10.7 mmHg	0.0051
RADIANCE-HTN SOLO	2018	Paradise ultrasonic catheter	2M	74	72	RHTN	RDN vs. sham	24-h SBP change	−7.0 ± 8.6 mmHg	−3.1 ± 9.7 mmHg	0.006
RADIANCE-HTN TRIO	2021	Paradise ultrasonic catheter	2M	69	67	RHTN	RDN vs. sham	DSBP change	−8.0 (−16.0 to 0.0) mmHg	−3.0 (−10.3 to 1.8) mmHg	0.022
Mahfound et al.	2020	Peregrine infusion catheter	6M	45	NA	NA	NA	24-h SBP change	−11 (−15 to −7) mmHg	NA	<0.001
ERADICATE-AF	2020	Irrigated-tip for PVI and RDN	12M	154	148	RHTN with AF	RDN + PVI vs. RDN	Freedom of AF	72.1%	56.5%	0.006
REACH Pilot	2013	Symplicity flex	6M	7	NA	RHTN with SHF	NA	6 MWT change	27.1 ± 9.7 m	NA	0.03
**PADN**											
PADN-1	2013	PADN catheter	3M	13	NA	IAPH	NA	MPAP change	−19 ± 5 mmHg	NA	<0.01
PADN-5	2019	PADN catheter	6M	48	50	Cpc-PH	PADN vs. sham	6 MWT change	83 m	15 m	<0.01
TROPHY-1	2020	TIVUS ultrasonic catheter	6M	20	NA	PH	NA	MPAP change	−5.1 ± 7.4 mmHg	NA	<0.01
**BAT**											
Rheos pivotal	2011	Rhoes system	6M	181	84	RHTN	Device on vs. off	Rate: SBP ≤ 140	42%	24%	0.005
Barostim neo	2012	Barostim neo system	6M	30	NA	RHTN	NA	OSBP change	−26.1 ± 3.3 mmHg	NA	<0.001
**EBA**											
CALM-FIM_EUR	2017	MobiusHD	6M	30	NA	RHTN	NA	OSBP change	−24 (−34 to −13) mmHg	NA	0.0003
**TCBA**											
Schlaich et al.	2020	Cibiem ultrasound transvenous system	6M	39	NA	RHTN	NA	24-h SBP change	−9.1 ± 13.5 mmHg	NA	NA
**SCS**											
SCS heart	2015	Eon mini system	6M	17	NA	SHF	NA	LVEF change	12%	NA	<0.01
DEFEAT-HF	2016	PrimeADVANCED neurostimulator	6M	42	24	SHF	Device on vs. off	LVESVi change	2.8	−3.6	0.30

*Data are presented as mean ± standard deviation or mean (95% confidential interval)*.

*RDN, renal denervation; PADN, pulmonary artery denervation; BAT, baroreflex activation therapy; EBA, endovascular baroreflex amplification; TCBA, transvenous carotid body ablation; SCS, spinal cord stimulation; M, month; EG, experimental group; NA, not applicable; RHTN, resistant hypertension; AF, atrial fibrillation; SHF, systolic heart failure; IPAH, idiopathic pulmonary artery hypertension; cpc-PH, combined pre- and post-capillary pulmonary hypertension; OSBP, office systolic blood pressure; SBP, systolic blood pressure; DSBP, daytime systolic blood pressure; 6 MWT, 6-min walking test; MPAP, mean pulmonary artery pressure; LVEF, left ventricular ejection fraction; LVESVi, left ventricular end systolic volume index*.

## Author Contributions

LL conceived the idea and wrote this manuscript. ZH and YLX collected the relevant articles. YY revised this manuscript. All authors have read and approved the final manuscript.

## Funding

This review was supported by National Natural Science Foundation of China (Grant Number: 81970285).

## Conflict of Interest

The authors declare that the research was conducted in the absence of any commercial or financial relationships that could be construed as a potential conflict of interest.

## Publisher's Note

All claims expressed in this article are solely those of the authors and do not necessarily represent those of their affiliated organizations, or those of the publisher, the editors and the reviewers. Any product that may be evaluated in this article, or claim that may be made by its manufacturer, is not guaranteed or endorsed by the publisher.

## References

[1] Malpas SC. Sympathetic nervous system overactivity and its role in the development of cardiovascular disease. Physiol Rev. (2010) 90:513–57. 10.1152/physrev.00007.2009 20393193

[2] Weldy CS Ashley EA. Towards precision medicine in heart failure. Nat Rev Cardiol. (2021) 18:745–62. 10.1038/s41569-021-00566-9 34108678 PMC12547831

[3] Lauder L Azizi M Kirtane AJ Bohm M Mahfoud F. Device-based therapies for arterial hypertension. Nat Rev Cardiol. (2020) 17:614–28. 10.1038/s41569-020-0364-1 32286512

[4] Huynh K. BP-lowering with renal denervation. Nat Rev Cardiol. (2020) 17:323. 10.1038/s41569-020-0380-1 32265546

[5] Fernandez-Ruiz I. Pulmonary artery denervation shows promise. Nat Rev Cardiol. (2020) 17:678. 10.1038/s41569-020-00442-y 32855534

[6] Victor RG. Carotid baroreflex activation therapy for resistant hypertension. Nat Rev Cardiol. (2015) 12:451–63. 10.1038/nrcardio.2015.96 26149485

[7] Huynh K. Heart failure: thoracic spinal cord stimulation for the treatment of HF. Nat Rev Cardiol. (2015) 12:66. 10.1038/nrcardio.2014.218 25560377

[8] Azizi M Sanghvi K Saxena M Gosse P Reilly JP Levy T. Ultrasound renal denervation for hypertension resistant to a triple medication pill (RADIANCE-HTN TRIO): a randomised, multicentre, single-blind, sham-controlled trial. Lancet. (2021) 397:2476–86. 10.1016/S0140-6736(21)00788-1 34010611

[9] Steinberg JS Shabanov V Ponomarev D Losik D Ivanickiy E Kropotkin E. Effect of renal denervation and catheter ablation vs. catheter ablation alone on atrial fibrillation recurrence among patients with paroxysmal atrial fibrillation and hypertension: the ERADICATE-AF randomized clinical trial. JAMA. (2020) 323:248–55. 10.1001/jama.2019.21187 31961420 PMC6990678

[10] Spadaro AG Bocchi EA Souza GE Filho AE Mariani J Jr. Campos CM. Renal denervation in patients with heart failure secondary to Chagas' disease: a pilot randomized controlled trial. Catheter Cardiovasc Interv. (2019) 94:644–50. 10.1002/ccd.28393 31334914

[11] Sata Y Head GA Denton K May CN Schlaich MP. Role of the sympathetic nervous system and its modulation in renal hypertension. Front Med (Lausanne). (2018) 5:82. 10.3389/fmed.2018.00082 29651418 PMC5884873

[12] Page IH Heuer GJ. The Effect of renal denervation on patients suffering from nephritis. J Clin Invest. (1935) 14:443–58. 10.1172/JCI100695 16694318 PMC424699

[13] DiBona GF Kopp UC. Neural control of renal function. Physiol Rev. (1997) 77:75–197. 10.1152/physrev.1997.77.1.75 9016301

[14] Kopp UC. Role of renal sensory nerves in physiological and pathophysiological conditions. Am J Physiol Regul Integr Comp Physiol. (2015) 308:R79–95. 10.1152/ajpregu.00351.2014 25411364 PMC4297860

[15] Fudim M Sobotka AA Yin YH Wang JW Levin H Esler M. Selective vs. global renal denervation: a case for less is more. Curr Hypertens Rep. (2018) 20:37. 10.1007/s11906-018-0838-2 29717380

[16] Norvell JE Anderson JM. Assessment of possible parasympathetic innervation of the kidney. J Auton Nerv Syst. (1983) 8:291–4. 10.1016/0165-1838(83)90112-1 6668390

[17] van Amsterdam WA Blankestijn PJ Goldschmeding R Bleys RL. The morphological substrate for renal denervation: nerve distribution patterns and parasympathetic nerves. A post-mortem histological study. Ann Anat. (2016) 204:71–9. 10.1016/j.aanat.2015.11.004 26617159

[18] de Jong MR Hoogerwaard AF Adiyaman A Smit JJJ Heeg JE van Hasselt B. Renal nerve stimulation identifies aorticorenal innervation and prevents inadvertent ablation of vagal nerves during renal denervation. Blood Press. (2018) 27:271–9. 10.1080/08037051.2018.1463817 29653494

[19] Liu H Chen W Lai Y Du H Wang Z Xu Y. Selective renal denervation guided by renal nerve stimulation in canine. Hypertension. (2019) 74:536–45. 10.1161/HYPERTENSIONAHA.119.12680 31327262

[20] de Jong MR Adiyaman A Gal P Smit JJ Delnoy PP Heeg JE. Renal nerve stimulation-induced blood pressure changes predict ambulatory blood pressure response after renal denervation. Hypertension. (2016) 68:707–14. 10.1161/HYPERTENSIONAHA.116.07492 27432864

[21] Hoogerwaard AF Adiyaman A de Jong MR Smit JJ Heeg JE van Hasselt B. Renal nerve stimulation: complete versus incomplete renal sympathetic denervation. Blood Press. (2021). 10.1080/08037051.2021.1982376. [Epub ahead of print]. 34647513

[22] Sakakura K Ladich E Cheng Q Otsuka F Yahagi K Fowler DR. Anatomic assessment of sympathetic peri-arterial renal nerves in man. J Am Coll Cardiol. (2014) 64:635–43. 10.1016/j.jacc.2014.03.059 25125292

[23] Garcia-Touchard A Maranillo E Mompeo B Sanudo JR. Microdissection of the human renal nervous system: implications for performing renal denervation procedures. Hypertension. (2020) 76:1240–6. 10.1161/HYPERTENSIONAHA.120.15106 32829660

[24] Bhatt DL Kandzari DE O'Neill WW D'Agostino R Flack JM Katzen BT. A controlled trial of renal denervation for resistant hypertension. N Engl J Med. (2014) 370:1393–401. 10.1056/NEJMoa1402670 24678939

[25] Fengler K Ewen S Hollriegel R Rommel KP Kulenthiran S Lauder L. Blood pressure response to main renal artery and combined main renal artery plus branch renal denervation in patients with resistant hypertension. J Am Heart Assoc. (2017) 6:196. 10.1161/JAHA.117.006196 28862930 PMC5586457

[26] Unger T Paulis L Sica DA. Therapeutic perspectives in hypertension: novel means for renin-angiotensin-aldosterone system modulation and emerging device-based approaches. Eur Heart J. (2011) 32:2739–47. 10.1093/eurheartj/ehr253 21951628 PMC3214724

[27] Machino T Murakoshi N Sato A Xu D Hoshi T Kimura T. Anti-hypertensive effect of radiofrequency renal denervation in spontaneously hypertensive rats. Life Sci. (2014) 110:86–92. 10.1016/j.lfs.2014.06.015 24984216

[28] Wang M Han W Zhang M Fang W Zhai X Guan S. Long-term renal sympathetic denervation ameliorates renal fibrosis and delays the onset of hypertension in spontaneously hypertensive rats. Am J Transl Res. (2018) 10:4042–53. 30662649 PMC6325514

[29] Mahfoud F Townsend RR Kandzari DE Kario K Schmieder RE Tsioufis K. Changes in plasma renin activity after renal artery sympathetic denervation. J Am Coll Cardiol. (2021) 77:2909–19. 10.1016/j.jacc.2021.04.044 33957242

[30] Roman RJ Cowley AW Jr. Characterization of a new model for the study of pressure-natriuresis in the rat. Am J Physiol. (1985) 248:F190–8. 10.1152/ajprenal.1985.248.2.F190 3970209

[31] Foss JD Fink GD Osborn JW. Reversal of genetic salt-sensitive hypertension by targeted sympathetic ablation. Hypertension. (2013) 61:806–11. 10.1161/HYPERTENSIONAHA.111.00474 23381790 PMC3658449

[32] Al-Khatib SM Stevenson WG Ackerman MJ Bryant WJ Callans DJ Curtis AB. 2017 AHA/ACC/HRS guideline for management of patients with ventricular arrhythmias and the prevention of sudden cardiac death: a report of the American College of Cardiology/American Heart Association Task Force on Clinical Practice Guidelines and the Heart Rhythm Society. J Am Coll Cardiol. (2018) 72:e91–e220. 10.1016/j.jacc.2017.10.053 29097296

[33] Nammas W Airaksinen JK Paana T Karjalainen PP. Renal sympathetic denervation for treatment of patients with atrial fibrillation: reappraisal of the available evidence. Heart Rhythm. (2016) 13:2388–94. 10.1016/j.hrthm.2016.08.043 27590432

[34] Huang B Yu L Scherlag BJ Wang S He B Yang K. Left renal nerves stimulation facilitates ischemia-induced ventricular arrhythmia by increasing nerve activity of left stellate ganglion. J Cardiovasc Electrophysiol. (2014) 25:1249–56. 10.1111/jce.12498 25066536

[35] Tsai WC Chan YH Chinda K Chen Z Patel J Shen C. Effects of renal sympathetic denervation on the stellate ganglion and brain stem in dogs. Heart Rhythm. (2017) 14:255–62. 10.1016/j.hrthm.2016.10.003 27720832 PMC5250538

[36] Sharp TE III Lefer DJ. Renal denervation to treat heart failure. Annu Rev Physiol. (2021) 83:39–58. 10.1146/annurev-physiol-031620-093431 33074771 PMC7878360

[37] Sharp TE III Polhemus DJ Li Z Spaletra P Jenkins JS Reilly JP. Renal denervation prevents heart failure progression *via* inhibition of the renin-angiotensin system. J Am Coll Cardiol. (2018) 72:2609–21. 10.1016/j.jacc.2018.08.2186 30466519 PMC12912457

[38] McMurray JJ Packer M Desai AS Gong J Lefkowitz MP Rizkala AR. Angiotensin-neprilysin inhibition versus enalapril in heart failure. N Engl J Med. (2014) 371:993–1004. 10.1056/NEJMoa1409077 25176015

[39] Polhemus DJ Trivedi RK Gao J Li Z Scarborough AL Goodchild TT. Renal sympathetic denervation protects the failing heart *via* inhibition of neprilysin activity in the kidney. J Am Coll Cardiol. (2017) 70:2139–53. 10.1016/j.jacc.2017.08.056 29050562

[40] Nishikimi T Maeda N Matsuoka H. The role of natriuretic peptides in cardioprotection. Cardiovasc Res. (2006) 69:318–28. 10.1016/j.cardiores.2005.10.001 16289003

[41] Morrissey DM Brookes VS Cooke WT. Sympathectomy in the treatment of hypertension; review of 122 cases. Lancet. (1953) 1:403–8. 10.1016/S0140-6736(53)91589-X 13024041

[42] Schlaich MP Sobotka PA Krum H Lambert E Esler MD. Renal sympathetic-nerve ablation for uncontrolled hypertension. N Engl J Med. (2009) 361:932–4. 10.1056/NEJMc0904179 19710497

[43] Krum H Schlaich M Whitbourn R Sobotka PA Sadowski J Bartus K. Catheter-based renal sympathetic denervation for resistant hypertension: a multicentre safety and proof-of-principle cohort study. Lancet. (2009) 373:1275–81. 10.1016/S0140-6736(09)60566-3 19332353

[44] Symplicity HTNI Esler MD Krum H Sobotka PA Schlaich MP Schmieder RE. Renal sympathetic denervation in patients with treatment-resistant hypertension (The Symplicity HTN-2 Trial): a randomised controlled trial. Lancet. (2010) 376:1903–9. 10.1016/S0140-6736(10)62039-9 21093036

[45] Mahfoud F Schmieder RE Azizi M Pathak A Sievert H Tsioufis C. Proceedings from the 2nd European Clinical Consensus Conference for device-based therapies for hypertension: state of the art and considerations for the future. Eur Heart J. (2017) 38:3272–81. 10.1093/eurheartj/ehx215 28475773 PMC5837218

[46] Azizi M Sapoval M Gosse P Monge M Bobrie G Delsart P. Optimum and stepped care standardised antihypertensive treatment with or without renal denervation for resistant hypertension (DENERHTN): a multicentre, open-label, randomised controlled trial. Lancet. (2015) 385:1957–65. 10.1016/S0140-6736(14)61942-5 25631070

[47] Lobo MD Sharp ASP Kapil V Davies J de Belder MA Cleveland T. Joint UK societies' 2019 consensus statement on renal denervation. Heart. (2019) 105:1456–63. 10.1136/heartjnl-2019-315098 31292190 PMC6817707

[48] Mahfoud F Bohm M Azizi M Pathak A Durand Zaleski I Ewen S. Proceedings from the European clinical consensus conference for renal denervation: considerations on future clinical trial design. Eur Heart J. (2015) 36:2219–27. 10.1093/eurheartj/ehv192 25990344

[49] Kiuchi MG Esler MD Fink GD Osborn JW Banek CT Bohm M. Renal denervation update from the international sympathetic nervous system summit: JACC state-of-the-art review. J Am Coll Cardiol. (2019) 73:3006–17. 10.1016/j.jacc.2019.04.015 31196459 PMC8559770

[50] Townsend RR Mahfoud F Kandzari DE Kario K Pocock S Weber MA. Catheter-based renal denervation in patients with uncontrolled hypertension in the absence of antihypertensive medications (SPYRAL HTN-OFF MED): a randomised, sham-controlled, proof-of-concept trial. Lancet. (2017) 390:2160–70. 10.1016/S0140-6736(17)32281-X 28859944

[51] Kandzari DE Bohm M Mahfoud F Townsend RR Weber MA Pocock S. Effect of renal denervation on blood pressure in the presence of antihypertensive drugs: 6-month efficacy and safety results from the SPYRAL HTN-ON MED proof-of-concept randomised trial. Lancet. (2018) 391:2346–55. 10.1016/S0140-6736(18)30951-6 29803589

[52] Kandzari DE Kario K Mahfoud F Cohen SA Pilcher G Pocock S. The SPYRAL HTN global clinical trial program: rationale and design for studies of renal denervation in the absence (SPYRAL HTN OFF-MED) and presence (SPYRAL HTN ON-MED) of antihypertensive medications. Am Heart J. (2016) 171:82–91. 10.1016/j.ahj.2015.08.021 26699604

[53] Bohm M Kario K Kandzari DE Mahfoud F Weber MA Schmieder RE. Efficacy of catheter-based renal denervation in the absence of antihypertensive medications (SPYRAL HTN-OFF MED Pivotal): a multicentre, randomised, sham-controlled trial. Lancet. (2020) 395:1444–51. 10.1016/S0140-6736(20)30554-7 32234534

[54] Azizi M Schmieder RE Mahfoud F Weber MA Daemen J Davies J. Endovascular ultrasound renal denervation to treat hypertension (RADIANCE-HTN SOLO): a multicentre, international, single-blind, randomised, sham-controlled trial. Lancet. (2018) 391:2335–45. 10.1016/S0140-6736(18)31082-1 29803590

[55] Azizi M Daemen J Lobo MD Mahfoud F Sharp ASP Schmieder RE. 12-Month results from the unblinded phase of the RADIANCE-HTN SOLO trial of ultrasound renal denervation. JACC Cardiovasc Interv. (2020) 13:2922–33. 10.1016/j.jcin.2020.09.054 33357531

[56] Kario K Yokoi Y Okamura K Fujihara M Ogoyama Y Yamamoto E. Catheter-based ultrasound renal denervation in patients with resistant hypertension: the randomized, controlled REQUIRE trial. Hypertens Res. (2021). 10.1038/s41440-021-00754-7. [Epub ahead of print]. 34654905 PMC8766280

[57] Fischell TA Ebner A Gallo S Ikeno F Minarsch L Vega F. Transcatheter alcohol-mediated perivascular renal denervation with the peregrine system: first-in-human experience. JACC Cardiovasc Interv. (2016) 9:589–98. 10.1016/j.jcin.2015.11.041 27013159

[58] Mahfoud F Renkin J Sievert H Bertog S Ewen S Bohm M. Alcohol-mediated renal denervation using the peregrine system infusion catheter for treatment of hypertension. JACC Cardiovasc Interv. (2020) 13:471–84. 10.1016/j.jcin.2019.10.048 32081241

[59] Mahfoud F Sievert H Bertog S Lauder L Ewen S Lengele JP. Long-term results up to 12 months after catheter-based alcohol-mediated renal denervation for treatment of resistant hypertension. Circ Cardiovasc Interv. (2021) 14:e010075. 10.1161/CIRCINTERVENTIONS.120.010075 34470501 PMC8452324

[60] Li Y Nawabi AQ Feng Y Dai Q Ma G Liu N. Safety and efficacy of a new renal denervation catheter in hypertensive patients in the absent of antihypertensive medications: a pilot study. Int J Hypertens. (2019) 2019:7929706. 10.1155/2019/7929706 30906590 PMC6393873

[61] Worthley SG Tsioufis CP Worthley MI Sinhal A Chew DP Meredith IT. Safety and efficacy of a multi-electrode renal sympathetic denervation system in resistant hypertension: the EnligHTN I trial. Eur Heart J. (2013) 34:2132–40. 10.1093/eurheartj/eht197 23782649 PMC3717311

[62] Wolf M Hubbard B Sakaoka A Rousselle S Tellez A Jiang X. Procedural and anatomical predictors of renal denervation efficacy using two radiofrequency renal denervation catheters in a porcine model. J Hypertens. (2018) 36:2453–9. 10.1097/HJH.0000000000001840 30005030 PMC6221386

[63] Sievert H Schofer J Ormiston J Hoppe UC Meredith IT Walters DL. Renal denervation with a percutaneous bipolar radiofrequency balloon catheter in patients with resistant hypertension: 6-month results from the REDUCE-HTN clinical study. EuroIntervention. (2015) 10:1213–20. 10.4244/EIJY14M12_01 25452197

[64] Verheye S Ormiston J Bergmann MW Sievert H Schwindt A Werner N. Twelve-month results of the rapid renal sympathetic denervation for resistant hypertension using the OneShotTM ablation system (RAPID) study. EuroIntervention. (2015) 10:1221–9. 10.4244/EIJY14M12_02 25452198

[65] Kim CJ Chang K Kim BK Park CG Jang Y. An open-label, single-arm, multicenter feasibility study evaluating the safety of catheter-based renal denervation with DENEX in patients with uncontrolled hypertension on standard medical therapy. Korean Circ J. (2021) 51:43–55. 10.4070/kcj.2020.0391 33377328 PMC7779817

[66] Cai X Yang Y Shen Y Wang W Qian L Cai J. Noninvasive stereotactic radiotherapy for renal denervation in a swine model. J Am Coll Cardiol. (2019) 74:1697–709. 10.1016/j.jacc.2019.07.053 31558254

[67] Fengler K Rommel KP Blazek S Besler C Hartung P von Roeder M. A three-arm randomized trial of different renal denervation devices and techniques in patients with resistant hypertension (RADIOSOUND-HTN). Circulation. (2019) 139:590–600. 10.1161/CIRCULATIONAHA.118.037654 30586691

[68] Schotten U Verheule S Kirchhof P Goette A. Pathophysiological mechanisms of atrial fibrillation: a translational appraisal. Physiol Rev. (2011) 91:265–325. 10.1152/physrev.00031.2009 21248168

[69] Fukuda K Kanazawa H Aizawa Y Ardell JL Shivkumar K. Cardiac innervation and sudden cardiac death. Circ Res. (2015) 116:2005–19. 10.1161/CIRCRESAHA.116.304679 26044253 PMC4465108

[70] Goldberger JJ Arora R Buckley U Shivkumar K. Autonomic nervous system dysfunction: JACC focus seminar. J Am Coll Cardiol. (2019) 73:1189–206. 10.1016/j.jacc.2018.12.064 30871703 PMC6958998

[71] Schwartz PJ Locati EH Moss AJ Crampton RS Trazzi R Ruberti U. Left cardiac sympathetic denervation in the therapy of congenital long QT syndrome. A worldwide report. Circulation. (1991) 84:503–11. 10.1161/01.CIR.84.2.503 1860195

[72] Kuhlkamp V Schirdewan A Stangl K Homberg M Ploch M Beck OA. Use of metoprolol CR/XL to maintain sinus rhythm after conversion from persistent atrial fibrillation: a randomized, double-blind, placebo-controlled study. J Am Coll Cardiol. (2000) 36:139–46. 10.1016/S0735-1097(00)00693-8 10898425

[73] Pokushalov E Romanov A Corbucci G Artyomenko S Baranova V Turov A. A randomized comparison of pulmonary vein isolation with versus without concomitant renal artery denervation in patients with refractory symptomatic atrial fibrillation and resistant hypertension. J Am Coll Cardiol. (2012) 60:1163–70. 10.1016/j.jacc.2012.05.036 22958958

[74] Schwartz PJ Priori SG Cerrone M Spazzolini C Odero A Napolitano C. Left cardiac sympathetic denervation in the management of high-risk patients affected by the long-QT syndrome. Circulation. (2004) 109:1826–33. 10.1161/01.CIR.0000125523.14403.1E 15051644

[75] Wilde AA Bhuiyan ZA Crotti L Facchini M De Ferrari GM Paul T. Left cardiac sympathetic denervation for catecholaminergic polymorphic ventricular tachycardia. N Engl J Med. (2008) 358:2024–9. 10.1056/NEJMoa0708006 18463378

[76] Bourke T Vaseghi M Michowitz Y Sankhla V Shah M Swapna N. Neuraxial modulation for refractory ventricular arrhythmias: value of thoracic epidural anesthesia and surgical left cardiac sympathetic denervation. Circulation. (2010) 121:2255–62. 10.1161/CIRCULATIONAHA.109.929703 20479150 PMC2896716

[77] Ukena C Bauer A Mahfoud F Schreieck J Neuberger HR Eick C. Renal sympathetic denervation for treatment of electrical storm: first-in-man experience. Clin Res Cardiol. (2012) 101:63–7. 10.1007/s00392-011-0365-5 21960416

[78] Ukena C Mahfoud F Ewen S Bollmann A Hindricks G Hoffmann BA. Renal denervation for treatment of ventricular arrhythmias: data from an International Multicenter Registry. Clin Res Cardiol. (2016) 105:873–9. 10.1007/s00392-016-1012-y 27364940

[79] Bradfield JS Hayase J Liu K Moriarty J Kee ST Do D. Renal denervation as adjunctive therapy to cardiac sympathetic denervation for ablation refractory ventricular tachycardia. Heart Rhythm. (2020) 17:220–7. 10.1016/j.hrthm.2019.09.016 31539629

[80] Normand C Kaye DM Povsic TJ Dickstein K. Beyond pharmacological treatment: an insight into therapies that target specific aspects of heart failure pathophysiology. Lancet. (2019) 393:1045–55. 10.1016/S0140-6736(18)32216-5 30860030

[81] Kaye DM Lambert GW Lefkovits J Morris M Jennings G Esler MD. Neurochemical evidence of cardiac sympathetic activation and increased central nervous system norepinephrine turnover in severe congestive heart failure. J Am Coll Cardiol. (1994) 23:570–8. 10.1016/0735-1097(94)90738-2 8113536

[82] Davies JE Manisty CH Petraco R Barron AJ Unsworth B Mayet J. First-in-man safety evaluation of renal denervation for chronic systolic heart failure: primary outcome from REACH-Pilot study. Int J Cardiol. (2013) 162:189–92. 10.1016/j.ijcard.2012.09.019 23031283

[83] Chen W Ling Z Xu Y Liu Z Su L Du H. Preliminary effects of renal denervation with saline irrigated catheter on cardiac systolic function in patients with heart failure: a Prospective, Randomized, Controlled, Pilot Study. Catheter Cardiovasc Interv. (2017) 89:E153–61. 10.1002/ccd.26475 27143319

[84] Gao JQ Yang W Liu ZJ. Percutaneous renal artery denervation in patients with chronic systolic heart failure: a randomized controlled trial. Cardiol J. (2019) 26:503–10. 10.5603/CJ.a2018.0028 29611171 PMC8084383

[85] Fukuta H Goto T Wakami K Kamiya T Ohte N. Effects of catheter-based renal denervation on heart failure with reduced ejection fraction: a meta-analysis of randomized controlled trials. Heart Fail Rev. (2020). 10.1007/s10741-020-09974-4. [Epub ahead of print]. 32394227

[86] Verloop WL Beeftink MM Santema BT Bots ML Blankestijn PJ Cramer MJ. A systematic review concerning the relation between the sympathetic nervous system and heart failure with preserved left ventricular ejection fraction. PLoS ONE. (2015) 10:e0117332. 10.1371/journal.pone.0117332 25658630 PMC4319815

[87] Valero-Munoz M Backman W Sam F. Murine models of heart failure with preserved ejection fraction: a “fishing expedition”. JACC Basic Transl Sci. (2017) 2:770–89. 10.1016/j.jacbts.2017.07.013 29333506 PMC5764178

[88] Riehle C Bauersachs J. Small animal models of heart failure. Cardiovasc Res. (2019) 115:1838–49. 10.1093/cvr/cvz161 31243437 PMC6803815

[89] Patel HC Rosen SD Hayward C Vassiliou V Smith GC Wage RR. Renal denervation in heart failure with preserved ejection fraction (RDT-PEF): a randomized controlled trial. Eur J Heart Fail. (2016) 18:703–12. 10.1002/ejhf.502 26990920

[90] Mahfoud F Urban D Teller D Linz D Stawowy P Hassel JH. Effect of renal denervation on left ventricular mass and function in patients with resistant hypertension: data from a multi-centre cardiovascular magnetic resonance imaging trial. Eur Heart J. (2014) 35:2224–31b. 10.1093/eurheartj/ehu093 24603307

[91] Veelken R Vogel EM Hilgers K Amann K Hartner A Sass G. Autonomic renal denervation ameliorates experimental glomerulonephritis. J Am Soc Nephrol. (2008) 19:1371–8. 10.1681/ASN.2007050552 18400940 PMC2440306

[92] Kiuchi MG Maia GL de Queiroz Carreira MA Kiuchi T Chen S Andrea BR. Effects of renal denervation with a standard irrigated cardiac ablation catheter on blood pressure and renal function in patients with chronic kidney disease and resistant hypertension. Eur Heart J. (2013) 34:2114–21. 10.1093/eurheartj/eht200 23786861

[93] Warchol-Celinska E Prejbisz A Kadziela J Florczak E Januszewicz M Michalowska I. Renal denervation in resistant hypertension and obstructive sleep apnea: randomized proof-of-concept phase II trial. Hypertension. (2018) 72:381–90. 10.1161/HYPERTENSIONAHA.118.11180 29941516

[94] Mahfoud F Schlaich M Kindermann I Ukena C Cremers B Brandt MC. Effect of renal sympathetic denervation on glucose metabolism in patients with resistant hypertension: a pilot study. Circulation. (2011) 123:1940–6. 10.1161/CIRCULATIONAHA.110.991869 21518978

[95] Zhang Z Liu K Xiao S Chen X. Effects of catheter-based renal denervation on glycemic control and lipid levels: a systematic review and meta-analysis. Acta Diabetol. (2021) 58:603–14. 10.1007/s00592-020-01659-6 33459896

[96] da Silva Goncalves Bos D Happe C Schalij I Pijacka W Paton JFR Guignabert C. Renal denervation reduces pulmonary vascular remodeling and right ventricular diastolic stiffness in experimental pulmonary hypertension. JACC Basic Transl Sci. (2017) 2:22–35. 10.1016/j.jacbts.2016.09.007 29034356 PMC5628179

[97] Lenski D Kindermann I Lenski M Ukena C Bunz M Mahfoud F. Anxiety, depression, quality of life and stress in patients with resistant hypertension before and after catheter-based renal sympathetic denervation. EuroIntervention. (2013) 9:700–8. 10.4244/EIJV9I6A114 24169132

[98] Bruno RM Taddei S Borghi C Colivicchi F Desideri G Grassi G. Italian Society of Arterial Hypertension (SIIA) position paper on the role of renal denervation in the management of the difficult-to-treat hypertensive patient. High Blood Press Cardiovasc Prev. (2020) 27:109–17. 10.1007/s40292-020-00367-0 32157642

[99] Booth LC Nishi EE Yao ST Ramchandra R Lambert GW Schlaich M. Reinnervation of renal afferent and efferent nerves at 55 and 11 months after catheter-based radiofrequency renal denervation in sheep. Hypertension. (2015) 65:393–400. 10.1161/HYPERTENSIONAHA.114.04176 25403610

[100] Mahfoud F Bohm M Schmieder R Narkiewicz K Ewen S Ruilope L. Effects of renal denervation on kidney function and long-term outcomes: 3-year follow-up from the Global SYMPLICITY Registry. Eur Heart J. (2019) 40:3474–82. 10.1093/eurheartj/ehz118 30907413 PMC6837160

[101] Hoogerwaard AF de Jong MR Adiyaman A Smit JJJ Delnoy P Heeg JE. Renal sympathetic denervation induces changes in heart rate variability and is associated with a lower sympathetic tone. Clin Res Cardiol. (2019) 108:22–30. 10.1007/s00392-018-1307-2 29943270

[102] Zhang W Zhang S Deng Y Wu S Ren J Sun G. Trial of intensive blood-pressure control in older patients with hypertension. N Engl J Med. (2021) 385:1268–79. 10.1056/NEJMoa2111437 34491661

[103] Pietzsch J Mahfoud F Williams B Mancia G Narkiewicz K Ruilope L. Clinical event reductions in high-risk hypertension patients treated with renal denervation: a model-based estimate based on 36-month data from the global symplicity registry. J Am Coll Cardiol. (2021) 77:1644. 10.1016/S0735-1097(21)03000-X33795039

[104] Schmieder RE Mahfoud F Mancia G Azizi M Bohm M Dimitriadis K. European Society of Hypertension position paper on renal denervation 2021. J Hypertens. (2021) 39:1733–41. 10.1097/HJH.0000000000002933 34261957

[105] Richardson JB. Nerve supply to the lungs. Am Rev Respir Dis. (1979) 119:785–802. 110183 10.1164/arrd.1979.119.5.785

[106] Huang Y Liu YW Pan HZ Zhang XL Li J Xiang L. Transthoracic pulmonary artery denervation for pulmonary arterial hypertension. Arterioscler Thromb Vasc Biol. (2019) 39:704–18. 10.1161/ATVBAHA.118.311992 30816802

[107] Barnes PJ Liu SF. Regulation of pulmonary vascular tone. Pharmacol Rev. (1995) 47:87–131. 7784481

[108] Rothman A Jonas M Castel D Tzafriri AR Traxler H Shav D. Pulmonary artery denervation using catheter-based ultrasonic energy. EuroIntervention. (2019) 15:722–30. 10.4244/EIJ-D-18-01082 31062694

[109] De Burgh Daly I Hebb C. Pulmonary vasomotor fibres in the cervical vagosympathetic nerve of the dog. Q J Exp Physiol Cogn Med Sci. (1952) 37:19–43. 10.1113/expphysiol.1952.sp000978 14912282

[110] Piene H. The influence of pulmonary blood flow rate on vascular input impedance and hydraulic power in the sympathetically and noradrenaline stimulated cat lung. Acta Physiol Scand. (1976) 98:44–53. 10.1111/j.1748-1716.1976.tb10301.x 970156

[111] Velez-Roa S Ciarka A Najem B Vachiery JL Naeije R van de Borne P. Increased sympathetic nerve activity in pulmonary artery hypertension. Circulation. (2004) 110:1308–12. 10.1161/01.CIR.0000140724.90898.D3 15337703

[112] Ameri P Bertero E Meliota G Cheli M Canepa M Brunelli C. Neurohormonal activation and pharmacological inhibition in pulmonary arterial hypertension and related right ventricular failure. Heart Fail Rev. (2016) 21:539–47. 10.1007/s10741-016-9566-3 27206576

[113] de Man FS Tu L Handoko ML Rain S Ruiter G Francois C. Dysregulated renin-angiotensin-aldosterone system contributes to pulmonary arterial hypertension. Am J Respir Crit Care Med. (2012) 186:780–9. 10.1164/rccm.201203-0411OC 22859525 PMC5104838

[114] Juratsch CE Jengo JA Castagna J Laks MM. Experimental pulmonary hypertension produced by surgical and chemical denervation of the pulmonary vasculature. Chest. (1980) 77:525–30. 10.1378/chest.77.4.525 7357977

[115] Chen SL Zhang FF Xu J Xie DJ Zhou L Nguyen T. Pulmonary artery denervation to treat pulmonary arterial hypertension: the single-center, prospective, first-in-man PADN-1 study (first-in-man pulmonary artery denervation for treatment of pulmonary artery hypertension). J Am Coll Cardiol. (2013) 62:1092–100. 10.1016/j.jacc.2013.05.075 23850902

[116] Chen SL Zhang H Xie DJ Zhang J Zhou L Rothman AM. Hemodynamic, functional, and clinical responses to pulmonary artery denervation in patients with pulmonary arterial hypertension of different causes: phase II results from the Pulmonary Artery Denervation-1 study. Circ Cardiovasc Interv. (2015) 8:e002837. 10.1161/CIRCINTERVENTIONS.115.002837 26553699 PMC4648183

[117] Pulido T Adzerikho I Channick RN Delcroix M Galie N Ghofrani HA. Macitentan and morbidity and mortality in pulmonary arterial hypertension. N Engl J Med. (2013) 369:809–18. 10.1056/NEJMoa1213917 23984728

[118] Zhang H Zhang J Chen M Xie DJ Kan J Yu W. Pulmonary artery denervation significantly increases 6-min walk distance for patients with combined pre- and post-capillary pulmonary hypertension associated with left heart failure: the PADN-5 study. JACC Cardiovasc Interv. (2019) 12:274–84. 10.1016/j.jcin.2018.09.021 30732732

[119] Romanov A Cherniavskiy A Novikova N Edemskiy A Ponomarev D Shabanov V. Pulmonary artery denervation for patients with residual pulmonary hypertension after pulmonary endarterectomy. J Am Coll Cardiol. (2020) 76:916–26. 10.1016/j.jacc.2020.06.064 32819465

[120] Rothman AMK Vachiery JL Howard LS Mikhail GW Lang IM Jonas M. Intravascular ultrasound pulmonary artery denervation to treat pulmonary arterial hypertension (TROPHY1): multicenter, early feasibility study. JACC Cardiovasc Interv. (2020) 13:989–99. 10.1016/j.jcin.2019.12.027 32327095

[121] Condori Leandro HI Koshevaya EG Mitrofanova LB Vakhrushev AD Goncharova NS Korobchenko LE. An ovine model for percutaneous pulmonary artery laser denervation: perivascular innervation and ablation lesion characteristics. Int J Mol Sci. (2021) 22:788. 10.3390/ijms22168788 34445490 PMC8395814

[122] Jiang YH Jiang P Yang JL Ma DF Lin HQ Su WG. Cardiac dysregulation and myocardial injury in a 6-hydroxydopamine-induced rat model of sympathetic denervation. PLoS ONE. (2015) 10:e0133971. 10.1371/journal.pone.0133971 26230083 PMC4521861

[123] Rosenkranz S Gibbs JS Wachter R De Marco T Vonk-Noordegraaf A Vachiery JL. Left ventricular heart failure and pulmonary hypertension. Eur Heart J. (2016) 37:942–54. 10.1093/eurheartj/ehv512 26508169 PMC4800173

[124] Galie N Humbert M Vachiery JL Gibbs S Lang I Torbicki A. 2015 ESC/ERS Guidelines for the diagnosis and treatment of pulmonary hypertension: the joint task force for the diagnosis and treatment of pulmonary hypertension of the European Society of Cardiology (ESC) and the European Respiratory Society (ERS): endorsed by: Association for European Paediatric and Congenital Cardiology (AEPC), International Society for Heart and Lung Transplantation (ISHLT). Eur Respir J. (2015) 46:903–75. 10.1183/13993003.01032-2015 26318161

[125] Yaylali YT Basarici I. Will pulmonary artery denervation really have a place in the armamentarium of the pulmonary hypertension specialist? JACC Cardiovasc Interv. (2019) 12:799–800. 10.1016/j.jcin.2019.02.015 31000018

[126] Goncharova NS Moiseeva OM Condori Leandro HI Zlobina IS Berezina AV Malikov KN. Electrical stimulation-guided approach to pulmonary artery catheter ablation in patients with idiopathic pulmonary arterial hypertension: a pilot feasibility study with a 12-month follow-up. Biomed Res Int. (2020) 2020:8919515. 10.1155/2020/8919515 32149144 PMC7048906

[127] Stirrup J Gregg S Baavour R Roth N Breault C Agostini D. Hybrid solid-state SPECT/CT left atrial innervation imaging for identification of left atrial ganglionated plexi: technique and validation in patients with atrial fibrillation. J Nucl Cardiol. (2020) 27:1939–50. 10.1007/s12350-018-01535-5 30694425

[128] Yi CX la Fleur SE Fliers E Kalsbeek A. The role of the autonomic nervous liver innervation in the control of energy metabolism. Biochim Biophys Acta. (2010) 1802:416–31. 10.1016/j.bbadis.2010.01.006 20060897

[129] Carnagarin R Kiuchi MG Goh G Adams L Cohen N Kavnoudias H. Role of the sympathetic nervous system in cardiometabolic control: implications for targeted multiorgan neuromodulation approaches. J Hypertens. (2021) 39:1478–89. 10.1097/HJH.0000000000002839 33657580

[130] Kraft G Vrba A Scott M Allen E Edgerton DS Williams PE. Sympathetic denervation of the common hepatic artery lessens glucose intolerance in the fat- and fructose-fed dog. Diabetes. (2019) 68:1143–55. 10.2337/db18-1209 30936143 PMC6610023

[131] Shimazu T Fukuda A. Increased activities of glycogenolytic enzymes in liver after splanchnic-nerve stimulation. Science. (1965) 150:1607–8. 10.1126/science.150.3703.1607 4286322

[132] Shimazu T Fukuda A Ban T. Reciprocal influences of the ventromedial and lateral hypothalamic nuclei on blood glucose level and liver glycogen content. Nature. (1966) 210:1178–9. 10.1038/2101178a0 5964188

[133] Buijs RM la Fleur SE Wortel J Van Heyningen C Zuiddam L Mettenleiter TC. The suprachiasmatic nucleus balances sympathetic and parasympathetic output to peripheral organs through separate preautonomic neurons. J Comp Neurol. (2003) 464:36–48. 10.1002/cne.10765 12866127

[134] Shimazu T. Central nervous system regulation of liver and adipose tissue metabolism. Diabetologia. (1981) 20:343–56. 10.1007/BF00254502 7014330

[135] Rojas JM Bruinstroop E Printz RL Alijagic-Boers A Foppen E Turney MK. Central nervous system neuropeptide Y regulates mediators of hepatic phospholipid remodeling and very low-density lipoprotein triglyceride secretion *via* sympathetic innervation. Mol Metab. (2015) 4:210–21. 10.1016/j.molmet.2015.01.004 25737956 PMC4338317

[136] Dicostanzo CA Dardevet DP Neal DW Lautz M Allen E Snead W. Role of the hepatic sympathetic nerves in the regulation of net hepatic glucose uptake and the mediation of the portal glucose signal. Am J Physiol Endocrinol Metab. (2006) 290:E9–E16. 10.1152/ajpendo.00184.2005 16105863

[137] Jackson PA Cardin S Coffey CS Neal DW Allen EJ Penaloza AR. Effect of hepatic denervation on the counterregulatory response to insulin-induced hypoglycemia in the dog. Am J Physiol Endocrinol Metab. (2000) 279:E1249–57. 10.1152/ajpendo.2000.279.6.E1249 11093911

[138] Kraft G Scott M Allen E Edgerton DS Farmer B Azamian BR. Safety of surgical denervation of the common hepatic artery in insulin-resistant dogs. Physiol Rep. (2021) 9:e14805. 10.14814/phy2.14805 33769710 PMC7995543

[139] Hurr C Simonyan H Morgan DA Rahmouni K Young CN. Liver sympathetic denervation reverses obesity-induced hepatic steatosis. J Physiol. (2019) 597:4565–80. 10.1113/JP277994 31278754 PMC6716997

[140] Carreno FR Seelaender MC. Liver denervation affects hepatocyte mitochondrial fatty acid transport capacity. Cell Biochem Funct. (2004) 22:9–17. 10.1002/cbf.1047 14695648

[141] Bruinstroop E Eliveld J Foppen E Busker S Ackermans MT Fliers E. Hepatic denervation and dyslipidemia in obese Zucker (fa/fa) rats. Int J Obes (Lond). (2015) 39:1655–8. 10.1038/ijo.2015.122 26134416

[142] Kiuchi MG Ganesan K Keating J Carnagarin R Matthews VB Herat LY. Combined renal and common hepatic artery denervation as a novel approach to reduce cardiometabolic risk: technical approach, feasibility and safety in a pre-clinical model. Clin Res Cardiol. (2021) 110:740–53. 10.1007/s00392-021-01814-1 33635438 PMC8099764

[143] Heusermann U Stutte HJ. Electron microscopic studies of the innervation of the human spleen. Cell Tissue Res. (1977) 184:225–36. 10.1007/BF00223070 922871

[144] Kudoh G Hoshi K Murakami T. Fluorescence microscopic and enzyme histochemical studies of the innervation of the human spleen. Arch Histol Jpn. (1979) 42:169–80. 10.1679/aohc1950.42.169 464750

[145] Guyot M Simon T Panzolini C Ceppo F Daoudlarian D Murris E. Apical splenic nerve electrical stimulation discloses an anti-inflammatory pathway relying on adrenergic and nicotinic receptors in myeloid cells. Brain Behav Immun. (2019) 80:238–46. 10.1016/j.bbi.2019.03.015 30885844

[146] Lewis SM Williams A Eisenbarth SC. Structure and function of the immune system in the spleen. Sci Immunol. (2019) 4:eaau6085. 10.1126/sciimmunol.aau6085 30824527 PMC6495537

[147] Whalley K. Brain-spleen link tunes immunity. Nat Rev Neurosci. (2020) 21:350–1. 10.1038/s41583-020-0316-0 32404989

[148] Zhang X Lei B Yuan Y Zhang L Hu L Jin S. Brain control of humoral immune responses amenable to behavioural modulation. Nature. (2020) 581:204–8. 10.1038/s41586-020-2235-7 32405000

[149] Rogausch H Del Rey A Kabiersch A Besedovsky HO. Interleukin-1 increases splenic blood flow by affecting the sympathetic vasoconstrictor tonus. Am J Physiol. (1995) 268:R902–8. 10.1152/ajpregu.1995.268.4.R902 7733400

[150] Franco M Tapia E Bautista R Pacheco U Santamaria J Quiroz Y. Impaired pressure natriuresis resulting in salt-sensitive hypertension is caused by tubulointerstitial immune cell infiltration in the kidney. Am J Physiol Renal Physiol. (2013) 304:F982–90. 10.1152/ajprenal.00463.2012 23364804 PMC3625854

[151] Carnevale D Perrotta M Pallante F Fardella V Iacobucci R Fardella S. A cholinergic-sympathetic pathway primes immunity in hypertension and mediates brain-to-spleen communication. Nat Commun. (2016) 7:13035. 10.1038/ncomms13035 27676657 PMC5052663

[152] Olofsson PS Katz DA Rosas-Ballina M Levine YA Ochani M Valdes-Ferrer SI. alpha7 nicotinic acetylcholine receptor (alpha7nAChR) expression in bone marrow-derived non-T cells is required for the inflammatory reflex. Mol Med. (2012) 18:539–43. 10.2119/molmed.2011.00405 22183893 PMC3356417

[153] Rosas-Ballina M Olofsson PS Ochani M Valdes-Ferrer SI Levine YA Reardon C. Acetylcholine-synthesizing T cells relay neural signals in a vagus nerve circuit. Science. (2011) 334:98–101. 10.1126/science.1209985 21921156 PMC4548937

[154] Borovikova LV Ivanova S Zhang M Yang H Botchkina GI Watkins LR. Vagus nerve stimulation attenuates the systemic inflammatory response to endotoxin. Nature. (2000) 405:458–62. 10.1038/35013070 10839541

[155] Tracey KJ. The inflammatory reflex. Nature. (2002) 420:853–9. 10.1038/nature01321 12490958

[156] Rosas-Ballina M Tracey KJ. The neurology of the immune system: neural reflexes regulate immunity. Neuron. (2009) 64:28–32. 10.1016/j.neuron.2009.09.039 19840545 PMC4533851

[157] Kooijman S Meurs I van Beek L Khedoe PP Giezekamp A Pike-Overzet K. Splenic autonomic denervation increases inflammatory status but does not aggravate atherosclerotic lesion development. Am J Physiol Heart Circ Physiol. (2015) 309:H646–54. 10.1152/ajpheart.00787.2014 26092978

[158] Kressel AM Tsaava T Levine YA Chang EH Addorisio ME Chang Q. Identification of a brainstem locus that inhibits tumor necrosis factor. Proc Natl Acad Sci USA. (2020) 117:29803–10. 10.1073/pnas.2008213117 33168718 PMC7703602

[159] Gautron L Rutkowski JM Burton MD Wei W Wan Y Elmquist JK. Neuronal and nonneuronal cholinergic structures in the mouse gastrointestinal tract and spleen. J Comp Neurol. (2013) 521:3741–67. 10.1002/cne.23376 23749724 PMC4081472

[160] Albaghdadi M Garcia-Polite F Zani B Keating J Melidone R Spognardi A. Splenic artery denervation: target micro-anatomy, feasibility, and early preclinical experience. Transl Res. (2019) 213:100–11. 10.1016/j.trsl.2019.07.012 31415732

[161] Sokal DM McSloy A Donega M Kirk J Colas RA Dolezalova N. Splenic nerve neuromodulation reduces inflammation and promotes resolution in chronically implanted pigs. Front Immunol. (2021) 12:649786. 10.3389/fimmu.2021.649786 33859641 PMC8043071

[162] Bronte V Pittet MJ. The spleen in local and systemic regulation of immunity. Immunity. (2013) 39:806–18. 10.1016/j.immuni.2013.10.010 24238338 PMC3912742

[163] Sagar S Liu PP Cooper LT Jr. Myocarditis. Lancet. (2012) 379:738–47. 10.1016/S0140-6736(11)60648-X 22185868 PMC5814111

[164] van Kleef M Bates MC Spiering W. Endovascular baroreflex amplification for resistant hypertension. Curr Hypertens Rep. (2018) 20:46. 10.1007/s11906-018-0840-8 29744599 PMC5942348

[165] Heusser K Tank J Brinkmann J Menne J Kaufeld J Linnenweber-Held S. Acute response to unilateral unipolar electrical carotid sinus stimulation in patients with resistant arterial hypertension. Hypertension. (2016) 67:585–91. 10.1161/HYPERTENSIONAHA.115.06486 26831195 PMC4773922

[166] Rothfeld EL Parsonnet V Raman KV Zucker IR Tiu R. The effect of carotid sinus nerve stimulation on cardiovascular dynamics in man. Angiology. (1969) 20:213–8. 10.1177/000331976902000405 4238072

[167] Illig KA Levy M Sanchez L Trachiotis GD Shanley C Irwin E. An implantable carotid sinus stimulator for drug-resistant hypertension: surgical technique and short-term outcome from the multicenter phase II Rheos feasibility trial. J Vasc Surg. (2006) 44:1213–8. 10.1016/j.jvs.2006.08.024 17145423

[168] Scheffers IJ Kroon AA Schmidli J Jordan J Tordoir JJ Mohaupt MG. Novel baroreflex activation therapy in resistant hypertension: results of a European multi-center feasibility study. J Am Coll Cardiol. (2010) 56:1254–8. 10.1016/j.jacc.2010.03.089 20883933

[169] Bisognano JD Bakris G Nadim MK Sanchez L Kroon AA Schafer J. Baroreflex activation therapy lowers blood pressure in patients with resistant hypertension: results from the double-blind, randomized, placebo-controlled rheos pivotal trial. J Am Coll Cardiol. (2011) 58:765–73. 10.1016/j.jacc.2011.06.008 21816315

[170] Wustmann K Kucera JP Scheffers I Mohaupt M Kroon AA de Leeuw PW. Effects of chronic baroreceptor stimulation on the autonomic cardiovascular regulation in patients with drug-resistant arterial hypertension. Hypertension. (2009) 54:530–6. 10.1161/HYPERTENSIONAHA.109.134023 19620513

[171] Heusser K Tank J Engeli S Diedrich A Menne J Eckert S. Carotid baroreceptor stimulation, sympathetic activity, baroreflex function, and blood pressure in hypertensive patients. Hypertension. (2010) 55:619–26. 10.1161/HYPERTENSIONAHA.109.140665 20101001

[172] de Leeuw PW Bisognano JD Bakris GL Nadim MK Haller H Kroon AA. Sustained reduction of blood pressure with baroreceptor activation therapy: results of the 6-year open follow-up. Hypertension. (2017) 69:836–43. 10.1161/HYPERTENSIONAHA.117.09086 28320856

[173] Hoppe UC Brandt MC Wachter R Beige J Rump LC Kroon AA. Minimally invasive system for baroreflex activation therapy chronically lowers blood pressure with pacemaker-like safety profile: results from the Barostim neo trial. J Am Soc Hypertens. (2012) 6:270–6. 10.1016/j.jash.2012.04.004 22694986

[174] Wachter R Halbach M Bakris GL Bisognano JD Haller H Beige J. An exploratory propensity score matched comparison of second-generation and first-generation baroreflex activation therapy systems. J Am Soc Hypertens. (2017) 11:81–91. 10.1016/j.jash.2016.12.003 28065708

[175] Wallbach M Born E Kampfer D Luders S Muller GA Wachter R. Long-term effects of baroreflex activation therapy: 2-year follow-up data of the BAT Neo system. Clin Res Cardiol. (2020) 109:513–22. 10.1007/s00392-019-01536-5 31388741

[176] Wallbach M Bohning E Lehnig LY Schroer C Muller GA Wachter R. Safety profile of baroreflex activation therapy (NEO) in patients with resistant hypertension. J Hypertens. (2018) 36:1762–9. 10.1097/HJH.0000000000001753 29677053

[177] Zile MR Lindenfeld J Weaver FA Zannad F Galle E Rogers T. Baroreflex activation therapy in patients with heart failure with reduced ejection fraction. J Am Coll Cardiol. (2020) 76:1–13. 10.1016/j.jacc.2020.05.015 32616150

[178] Spiering W Williams B Van der Heyden J van Kleef M Lo R Versmissen J. Endovascular baroreflex amplification for resistant hypertension: a safety and proof-of-principle clinical study. Lancet. (2017) 390:2655–61. 10.1016/S0140-6736(17)32337-1 28870716

[179] Kara T Narkiewicz K Somers VK. Chemoreflexes–physiology and clinical implications. Acta Physiol Scand. (2003) 177:377–84. 10.1046/j.1365-201X.2003.01083.x 12609009

[180] Schultz HD Li YL Ding Y. Arterial chemoreceptors and sympathetic nerve activity: implications for hypertension and heart failure. Hypertension. (2007) 50:6–13. 10.1161/HYPERTENSIONAHA.106.076083 17502495

[181] Sinski M Lewandowski J Przybylski J Bidiuk J Abramczyk P Ciarka A. Tonic activity of carotid body chemoreceptors contributes to the increased sympathetic drive in essential hypertension. Hypertens Res. (2012) 35:487–91. 10.1038/hr.2011.209 22158114

[182] Narkiewicz K Ratcliffe LE Hart EC Briant LJ Chrostowska M Wolf J. Unilateral carotid body resection in resistant hypertension: a safety and feasibility trial. JACC Basic Transl Sci. (2016) 1:313–24. 10.1016/j.jacbts.2016.06.004 27766316 PMC5063532

[183] Schlaich M Sievert H Reddy V Hering D Schultz C Shetty S. First-in-human evaluation of a transvenous carotid body ablation device to treat patients with resistant hypertension. J Hypertens. (2017) 35:e64. 10.1097/01.hjh.0000523143.97845.cc

[184] Schlaich M Schultz C Shetty S Hering D Worthley S Delacroix S. Transvenous carotid body ablation for resistant hypertension: main results of a multicentre safety and proof-of-principle cohort study. Eur Heart J. (2018) 39:1416. 10.1093/eurheartj/ehy565.141629300883

[185] Aarabi B. Personalising pain control with spinal cord stimulation. Lancet Neurol. (2020) 19:103–4. 10.1016/S1474-4422(19)30484-3 31870767

[186] Howard-Quijano K Takamiya T Dale EA Kipke J Kubo Y Grogan T. Spinal cord stimulation reduces ventricular arrhythmias during acute ischemia by attenuation of regional myocardial excitability. Am J Physiol Heart Circ Physiol. (2017) 313:H421–31. 10.1152/ajpheart.00129.2017 28576833 PMC5582923

[187] Anselmino M Ravera L De Luca A Capriolo M Bordese R Trevi GP. Spinal cord stimulation and 30-minute heart rate variability in refractory angina patients. Pacing Clin Electrophysiol. (2009) 32:37–42. 10.1111/j.1540-8159.2009.02174.x 19140911

[188] Naar J Jaye D Linde C Neuzil P Doskar P Malek F. Effects of spinal cord stimulation on cardiac sympathetic nerve activity in patients with heart failure. Pacing Clin Electrophysiol. (2017) 40:504–13. 10.1111/pace.13050 28206674

[189] Ekre O Eliasson T Norrsell H Wahrborg P Mannheimer C Electrical Stimulation versus Coronary Artery Bypass Surgery in Severe Angina Pectoris. Long-term effects of spinal cord stimulation and coronary artery bypass grafting on quality of life and survival in the ESBY study. Eur Heart J. (2002) 23:1938–45. 10.1053/euhj.2002.3286 12473256

[190] Tse HF Turner S Sanders P Okuyama Y Fujiu K Cheung CW. Thoracic spinal cord stimulation for heart failure as a restorative treatment (SCS HEART study): first-in-man experience. Heart Rhythm. (2015) 12:588–95. 10.1016/j.hrthm.2014.12.014 25500165

[191] Zipes DP Neuzil P Theres H Caraway D Mann DL Mannheimer C. Determining the feasibility of spinal cord neuromodulation for the treatment of chronic systolic heart failure: the DEFEAT-HF study. JACC Heart Fail. (2016) 4:129–36. 10.1016/j.jchf.2015.10.006 26682789

[192] Issa ZF Zhou X Ujhelyi MR Rosenberger J Bhakta D Groh WJ. Thoracic spinal cord stimulation reduces the risk of ischemic ventricular arrhythmias in a postinfarction heart failure canine model. Circulation. (2005) 111:3217–20. 10.1161/CIRCULATIONAHA.104.507897 15956128

[193] Lopshire JC Zhou X Dusa C Ueyama T Rosenberger J Courtney N. Spinal cord stimulation improves ventricular function and reduces ventricular arrhythmias in a canine postinfarction heart failure model. Circulation. (2009) 120:286–94. 10.1161/CIRCULATIONAHA.108.812412 19597055

[194] Odenstedt J Linderoth B Bergfeldt L Ekre O Grip L Mannheimer C. Spinal cord stimulation effects on myocardial ischemia, infarct size, ventricular arrhythmia, and noninvasive electrophysiology in a porcine ischemia-reperfusion model. Heart Rhythm. (2011) 8:892–8. 10.1016/j.hrthm.2011.01.029 21255678

[195] Grimaldi R de Luca A Kornet L Castagno D Gaita F. Can spinal cord stimulation reduce ventricular arrhythmias? Heart Rhythm. (2012) 9:1884–7. 10.1016/j.hrthm.2012.08.007 22877745

[196] Downar J Blumberger DM Daskalakis ZJ. Repetitive transcranial magnetic stimulation: an emerging treatment for medication-resistant depression. CMAJ. (2016) 188:1175–7. 10.1503/cmaj.151316 27551033 PMC5088079

[197] Wang S Zhou X Huang B Wang Z Zhou L Wang M. Noninvasive low-frequency electromagnetic stimulation of the left stellate ganglion reduces myocardial infarction-induced ventricular arrhythmia. Sci Rep. (2016) 6:30783. 10.1038/srep30783 27470078 PMC4965791

[198] Markman TM Hamilton RH Marchlinski FE Nazarian S. Case series of transcutaneous magnetic stimulation for ventricular tachycardia storm. JAMA. (2020) 323:2200–2. 10.1001/jama.2020.3833 32372071 PMC7201374

[199] Wang S Li B Li X Wu L Zhu T Zhao D. Low-intensity ultrasound modulation may prevent myocardial infarction-induced sympathetic neural activation and ventricular arrhythmia. J Cardiovasc Pharmacol. (2020) 75:432–8. 10.1097/FJC.0000000000000810 32079857

[200] Yu L Scherlag BS Dormer K Rutel I Huang B Zhou X. Targeted ganglionated plexi denervation using magnetic nanoparticles carrying calcium chloride payload. JACC Clin Electrophysiol. (2018) 4:1347–58. 10.1016/j.jacep.2018.06.012 30336881 PMC6598434

